# AWmeta Empowers Adaptively Weighted Transcriptomic Meta-Analysis

**DOI:** 10.3390/cimb48050530

**Published:** 2026-05-19

**Authors:** Yanshi Hu, Zixuan Wang, Yueming Hu, Cong Feng, Qiuyu Fang, Ming Chen

**Affiliations:** 1 Department of Bioinformatics, College of Life Sciences, Zhejiang University, Hangzhou 310058, China; yanshihu@zju.edu.cn (Y.H.); zixuan.wang@zju.edu.cn (Z.W.); huym@fynu.edu.cn (Y.H.); ventson@zju.edu.cn (C.F.); 2 Institute of Hematology, Zhejiang University School of Medicine, Hangzhou 310058, China; 3 State Key Laboratory of Transvascular Implantation Devices, Zhejiang University School of Medicine, Hangzhou 310058, China; 3180104055@zju.edu.cn

**Keywords:** transcriptomic meta-analysis, random-effects model, differentially expressed gene, disease gene prioritization, functional enrichment

## Abstract

Transcriptomic meta-analysis enhances biological veracity and reproducibility of differentially expressed genes (DEGs) by integrating multiple independent studies, yet prevailing *p*-value or effect-size integration approaches exhibit limited power to resolve subtle yet vital gene signatures. This study presents AWmeta, an adaptively weighted framework that unifies both meta-analytical paradigms for the first time. Benchmarked on 35 Parkinson’s and Crohn’s disease datasets spanning diverse tissues and adaptively down-weighting underpowered studies, AWmeta yields higher-fidelity DEGs with markedly reduced false positives and achieves more truthful gene differential expression quantification across individual studies at both gene and study levels over the random-effects model (REM). Resilience experiments demonstrate AWmeta’s remarkable stability and robustness against external and internal perturbations. Crucially, AWmeta prioritizes more tissue-contextual genes of Parkinson’s and Crohn’s disease with genuine pathological importance than those from REM and constituent studies. Functional enrichment analysis further verifies that these screened gene signatures capture higher contextual coherence in all analyzed disease tissues. AWmeta harmonizes heterogeneous transcriptomic datasets into reliable DEG identification and mechanistic insights, serving as an indispensable tool for precision transcriptomic integration.

## 1. Introduction

The exponential expansion of publicly available transcriptomic data, propelled by high-throughput sequencing advancements [1], presents unprecedented opportunities and concomitant challenges for uncovering robust biological insights through meta-analysis. By integrating findings across independent studies, this powerful approach transcends the limitations of individual datasets, mitigating issues of statistical power, experimental variability, tissue heterogeneity, and platform-specific biases that often obscure subtle yet pathologically relevant expression signatures [2,3]. As complex diseases increasingly defy dissection by single-study designs, meta-analysis has become indispensable for identifying reproducible biomarkers and elucidating disease pathways with enhanced confidence and precision [4,5].

Transcriptomic meta-analysis demands both high statistical confidence in identifying dysregulated genes and accurate quantitative estimates of their expression changes [6,7]. Current *p*-value integration schemes, e.g., Fisher’s [8], Stouffer’s Z-score [9], and AW-Fisher [10], while adept at pinpointing consistently altered genes, offer minimal information on the magnitude or biological relevance of these alterations. Conversely, effect-size methods, exemplified by the random-effects model (REM) [11], though designed to quantify these changes, struggle with the pervasive heterogeneity inherent in pooling diverse experimental designs, tissue sources, or patient cohorts, potentially diminishing the estimate reliability [12]. Rank-based techniques, e.g., RankProd and RankSum [13,14], offer robustness against outliers but often at the cost of statistical resolution and power. Despite its advantages in handling study heterogeneity, Bayesian meta-analysis carries inherent limitations, including potential subjectivity in prior specification, mandatory Markov chain Monte Carlo convergence validation, and the absence of unified reporting standards [15,16]. Furthermore, these distinct methodological frameworks typically operate in isolation, which fails to synergistically leverage their complementary strengths, thereby limiting the overall sensitivity and the depth of achievable insights.

To address these critical gaps, we introduce AWmeta, a novel transcriptomic meta-analytic approach that unifies the statistical rigor of the *p*-value–based method with the quantitative power of the effect-size paradigm. Validated on 35 diverse transcriptomic datasets spanning Parkinson’s and Crohn’s disease across multiple tissues, AWmeta consistently outperforms the state-of-the-art REM. It secures higher-fidelity differentially expressed gene (DEG) identification with markedly reduced false positives, exhibits more truthful gene differential expression quantification across individual studies, and maintains remarkable stability and robustness against various perturbations. More importantly, AWmeta screens tissue-contextual disease genes with significantly greater overlap with three different disease gene benchmark datasets. Functional enrichment analysis of these prioritized genes further captures tissue-wise pathological mechanisms of Parkinson’s and Crohn’s disease with higher coherence. Therefore, with public transcriptomic repositories such as Gene Expression Omnibus (GEO) and Sequence Read Archive (SRA) experiencing sustained exponential growth over the past two decades [1,17], AWmeta empowers researchers to mine this rapidly accumulating data resource more effectively, accelerating the discovery of actionable molecular insights across biomedical domains.

## 2. Materials and Methods

### 2.1. Framework of AWmeta

The core innovation of AWmeta lies in an adaptively weighting scheme that identifies and up-weights the most informative transcriptomic studies while robustly mitigating noise and outliers to yield biologically coherent and high-fidelity meta-analytic estimates.

Well-established meta-analysis effect size estimators such as Cohen’s *d* [18] or Hedges’ *g* [19] are constrained by standard deviation, which might be biased in a transcriptomic scenario. For example, within RNA-sequencing (RNA-seq) data, highly expressed housekeeping genes often have low variance, inflating Cohen’s *d* or Hedges’ *g* despite small biological change. Therefore, in the AWmeta architecture, fold change is used to estimate effect size, which has been widely used and proven effective as a demonstrably reliable effect-size metric [20] in meta-analyses that integrate transcriptomic or proteomic studies comparing disease versus control conditions [21,22,23].

**Figure 1:**
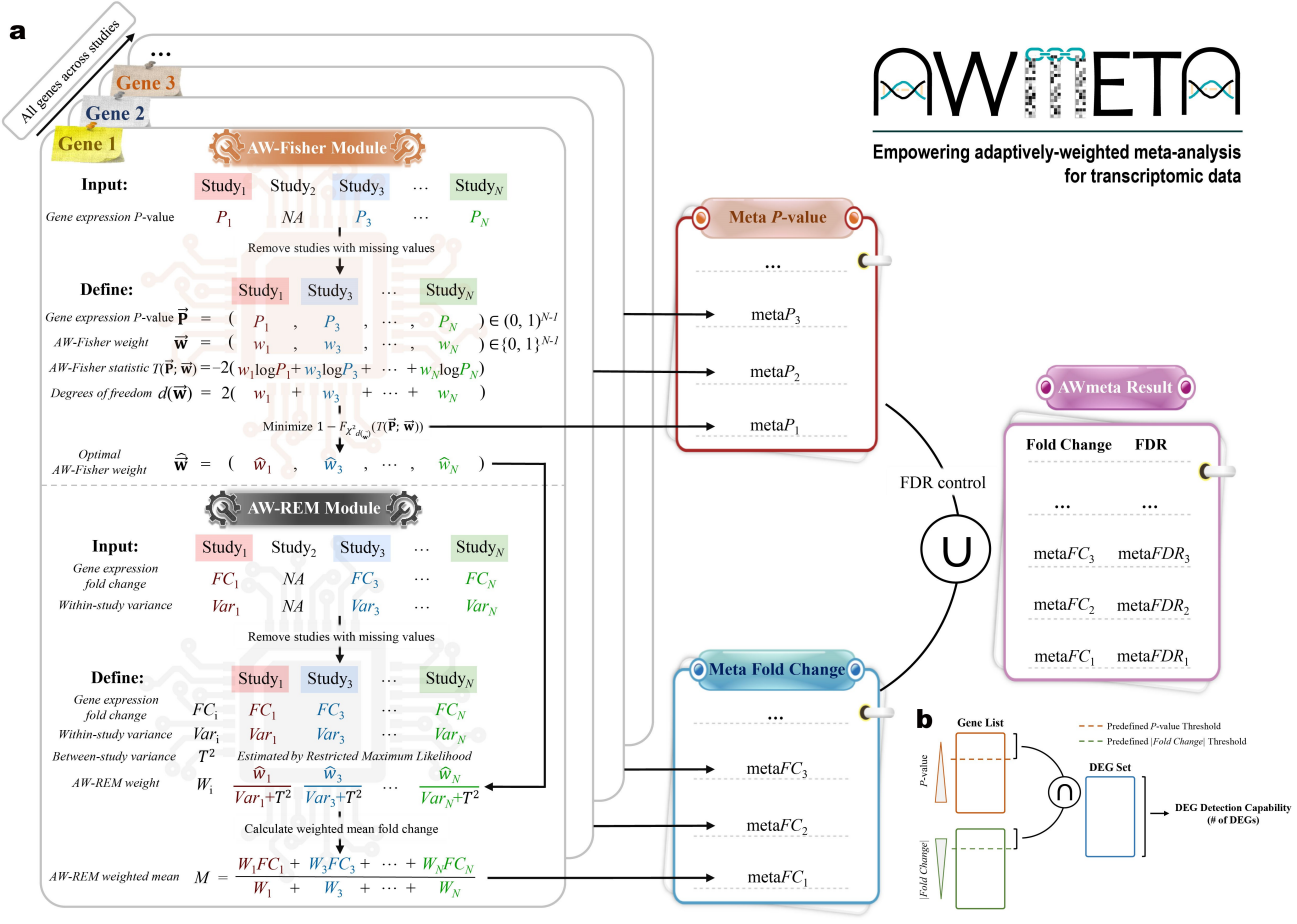

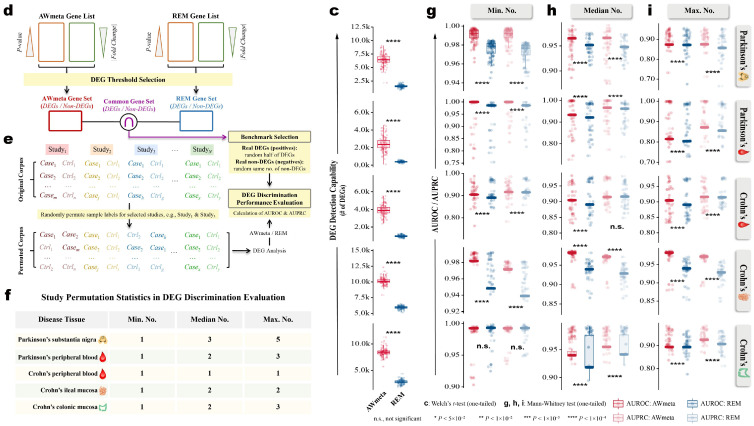
Overview of AWmeta and DEG identification evaluation in Parkinson’s and Crohn’s disease contexts. (**a**) Schematic of the AWmeta framework. (**b**) Schematic of DEG detection capability. (**c**) DEG detection capability performance comparisons between AWmeta and REM with corrected *p*-value (FDR) < 0.01 and fold change (|log_2_FC|) > log_2_1.2 across five disease tissues. Statistical significance was determined with one-tailed Welch’s *t*-test. (**d**) Strategy for generating the semi-synthetic benchmark dataset, sampling equivalent DEGs and non-DEGs from common genes identified by both AWmeta and REM. (**e**) Workflow for evaluating DEG discrimination performance using sample label permutation within the semi-synthetic benchmark dataset, followed by AWmeta/REM procedure and AUROC/AUPRC calculation. (**f**) Study permutation statistics (number of permuted studies) in the DEG discrimination evaluation procedure across five disease tissues. (**g**–**i**) DEG discrimination performance comparisons between AWmeta and REM using minimum-, median-, and maximum-permuted semi-synthetic simulation strategy with FDR < 0.01 and |log_2_FC| > log_2_1.2 across five disease tissues. Statistical significance was determined using a one-tailed Mann–Whitney test. Boxplot bounds indicate interquartile ranges (IQR), centers denote median values, and whiskers extend to 1.5 × IQR. The following icons represent different tissue sources: 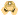
—substantia nigra; 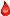
—peripheral blood; 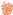
—ileal mucosa; and 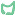
—colonic mucosa. n.s., not significant. *, $p<5\times 10^{-2}$. **, $p<1\times 10^{-2}$. ***, $p<1\times 10^{-3}$. ****, $p<1\times 10^{-4}$.

This framework performs a gene-by-gene meta-analysis of heterogeneous transcriptomic studies by integrating per-study gene summary statistics (Figure 1a). For each gene, only those studies are included in the valid set ($S_{gene}$) for subsequent meta-analysis of the gene that report a valid *p*-value ($P_{i}$), log_2_-based fold change ($FC_{i}$), and its corresponding within-study variance ($Var_{i}$), all derived from the original gene differential expression analyses. For each original study, no imputation procedure for gene expression missing values was performed. Studies absent from $S_{gene}$ (e.g., Study_2_ with missing $P_{2}$, $FC_{2}$, or $Var_{2}$) are excluded a priori. AWmeta consists of two sequential modules: AW-Fisher for adaptive *p*-value aggregation and AW-REM for adaptive effect-size integration. The AW-Fisher module yields optimal study-specific weights that indicate which subset of studies produces the most statistically significant combined probability; subsequently, in the AW-REM module, these optimized weights are embedded into the REM architecture to derive weighted fold change estimates.

#### 2.1.1. AW-Fisher Module (Adaptive *p*-Value Integration)

Within this module, each gene’s meta *p*-value is obtained by selecting an optimal subset of the valid transcriptomic set $S_{gene}$ that minimizes a weighted Fisher’s statistic-derived combined *p*-value [10]. Let $N^{\prime }=\left|S_{gene}\right|$ be the number of studies reporting *p*-values for the gene, with $S_{gene}^{\prime }=\left\{1,\ldots ,N^{\prime }\right\}$ enumerating the study indices, and denote their *p*-values by $\vec{P}={\left(P_{1},\ldots ,P_{i}\right)}_{i\in S_{gene}^{\prime }}\in {\left(0,1\right)}^{N^{\prime }}$. The corresponding binary weight vector, $\vec{w}={\left(w_{1},\ldots ,w_{i}\right)}_{i\in S_{gene}^{\prime }}\in {\left\{0,1\right\}}^{N^{\prime }}$, indicates inclusion ($w_{i}=1$) or exclusion ($w_{i}=0$) of Study_*i*_ $\in S_{gene}$ in the final subset. The AW-Fisher statistic is defined as:

(1) $$T\left(\vec{P};\vec{w}\right)=-2\sum_{i\in S_{gene}^{\prime }}w_{i}\ln P_{i}$$

The significance level of $T(\vec{P};\vec{w})$ under the null hypothesis is calculated using the chi-squared distribution:

(2) $$L\left(T\left(\vec{P};\vec{w}\right)\right)=1-F_{\chi_{d(\vec{w})}^{2}}\left(T\left(\vec{P};\vec{w}\right)\right)$$

where the degrees of freedom are $d\left(\vec{w}\right)=2\sum_{i\in S_{gene}^{\prime }}w_{i}$, and $F_{\chi_{d}^{2}}\left(\cdot \right)$ is the cumulative distribution function of the chi-squared distribution with *d* degrees of freedom.

The meta *p*-value, $s(\vec{P})$, is the minimum significance level obtained by optimizing the weight vector over the studies in $S_{gene}$:

(3) $$s\left(\vec{P}\right)=\min_{\vec{w}}\;L\left(T\left(\vec{P};\vec{w}\right)\right)$$

The optimal weight vector $\hat{w}$ that achieves this minimum is determined by

(4) $$\hat{w}=w\left(\vec{P}\right)=\underset{\vec{w}}{\mathrm{argmin}}\;L\left(T\left(\vec{P};\vec{w}\right)\right)={\left({\hat{w}}_{1},\ldots ,{\hat{w}}_{i}\right)}_{i\in S_{gene}^{\prime }}$$

This optimal weight vector $\hat{w}$, containing binary weights for each study in $S_{gene}$, is passed to the following AW-REM module.

#### 2.1.2. AW-REM Module (Adaptive Effect-Size Integration)

This module calculates the meta effect size (log_2_FC) using an adaptively weighted REM. It leverages the log_2_FC ($FC_{i}$) and within-study variance ($Var_{i}$) from studies in $S_{gene}$, modulated by the optimal binary weights $\hat{w}$ derived from the AW-Fisher module for those same studies. The contribution weight for Study*_i_* $\in S_{gene}$ in AW-REM is defined as

(5) $$W_{i}=\frac{{\hat{w}}_{i}}{Var_{i}+T^{2}}$$

where ${\hat{w}}_{i}$ is the binary weight (0 or 1) for Study*_i_* $\in S_{gene}$ from Equation (4). $Var_{i}$ is the within-study variance for the gene in Study*_i_*, and $T^{2}$ represents the sample estimate of between-study variance, measured using the restricted maximum likelihood (REML) method [24]. $W_{i}$ is zero whenever ${\hat{w}}_{i}=0$, thus automatically omitting studies not selected by the AW-Fisher module.

The final meta fold change estimate, denoted M, is computed as an adaptively calibrated average of the study-wise effect sizes:

(6) $$M=\frac{\sum_{i\in S_{gene}^{\prime }}W_{i}FC_{i}}{\sum_{i\in S_{gene}^{\prime }}W_{i}}$$

This formulation delivers a consensus fold-change estimate both statistically rigorous and quantitatively faithful to the most informative subsets of heterogeneous studies.

### 2.2. Transcriptomic Datasets

To provide a proof-of-concept for the AWmeta framework, 35 publicly available human transcriptomic datasets for Parkinson’s and Crohn’s disease were compiled from GEO, SRA, and ArrayExpress. These datasets, encompassing both microarray and RNA-seq platforms, included samples derived from Parkinson’s substantia nigra and peripheral blood, Crohn’s peripheral blood, and ileal and colonic mucosa. A complete list of the datasets, detailing data accession IDs, sequencing platform identifiers, dataset and tissue sources, and patient and control sample sizes, is provided in Supplementary Figure S1.

### 2.3. Transcriptomic Data Preprocessing

Due to the inclusion of datasets generated on different platforms, specific preprocessing pipelines were applied separately to microarray and RNA-seq data.

#### 2.3.1. Microarray Data Processing

To ensure accurate and up-to-date probe annotations, microarray probe identifiers were mapped to Entrez Gene IDs using information retrieved from GEO SOFT files, platform-specific Bioconductor annotation packages, and the AnnoProbe R package (version 0.1.8). For genes represented by multiple probes, the probe with the largest interquartile range (IQR) of intensities across samples was retained to maximize biological informativeness [25,26]. Subsequently, the limma R package [27] was utilized for microarray data preprocessing, normalization, and differential gene identification. Gene differential expression (case versus control) within each study was determined via empirical Bayes moderated *t*-statistics, yielding per-gene $P_{i}$, $FC_{i}$, and $Var_{i}$.

#### 2.3.2. RNA-Seq Data Processing

RNA-seq data were uniformly processed through an automated snakemake workflow [28]. Raw sequencing reads were processed with Trimmomatic [29] to remove adapter sequences and low-quality bases. Following best practice recommendations [30], the cleaned reads were aligned to the human reference genome (GRCh38 assembly) using HISAT2 [31]. Gene-level read counts were quantified from the aligned reads with featureCounts [32]. Finally, gene differential expression quantification was performed using DESeq2 with variance-stabilizing transformation [20], producing per-gene $P_{i}$, $FC_{i}$, and $Var_{i}$ for each study.

### 2.4. Transcriptomic Meta-Analysis Evaluation Metrics

To impartially evaluate AWmeta’s performance advances, a multi-dimensional comparison was conducted against the current gold-standard REM method [25,33,34] across the following critical domains: (i) DEG detection capability, (ii) DEG discrimination, (iii) gene- and study-wise gene differential expression convergence, (iv) stability and robustness, and (v) biological relevance. Both methods operated on matching inputs and identical gene sets, ensuring an equitable performance assessment.

#### 2.4.1. DEG Detection Capability Evaluation

DEG detection capability is defined as the gene count satisfying pre-defined thresholds for both corrected statistical significance *p*-value (FDR) and fold change magnitude (|log_2_FC|) (Figure 1b). To assess the stability and reliability of this capability, a bootstrap resampling strategy with 100 iterations was implemented. In each iteration, bootstrapped datasets were created via randomly sampling with replacement from the original case and control groups while maintaining the original sample sizes, followed by meta-analysis. The resulting DEG counts formed a distribution for statistical comparison with one-tailed Welch’s *t*-test.

#### 2.4.2. DEG Discrimination Evaluation Using Semi-Synthetic Simulation Strategy

To evaluate the ability to discriminate between DEGs and non-DEGs, particularly considering potential false positives arising from higher detection sensitivity, an evaluation metric based on semi-synthetic simulated data was adopted, inspired by Li and colleagues [35]. This approach consisted of benchmark dataset generation and evaluation using datasets with simulated noise (Figure 1d–f) and for each tissue context:

1. Identify the intersection of DEGs and non-DEGs called by both AWmeta and REM under predefined screening thresholds (Figure 1d).
2. Randomly sample half of the intersected DEGs to form an unbiased positive benchmark; sample an equal-sized negative benchmark from the intersected non-DEGs (Figure 1d).
3. Construct semi-synthetic datasets by permuting case/control labels within a subset of original studies (e.g., Study_1_ and Study_3_; Figure 1e). Label permutation removes the true signal from those studies.
4. Apply AWmeta and REM to the combined set of original and label-permuted studies; compute the area under the receiver operating characteristic curve (AUROC) and the area under the precision–recall curve (AUPRC) over the previously defined positive and negative benchmark genes (Figure 1e).
5. Repeat Steps 3 and 4 100 times to obtain distributions of AUROC and AUPRC, summarizing performance under minimum-, median-, and maximum-permuted scenarios (Figure 1f), which ensures the stability and reliability of our assessment. Statistical significance between AWmeta and REM was tested via a one-tailed Mann–Whitney test.

#### 2.4.3. Gene-Wise Convergence Assessment for Gene Differential Expression Meta-Analysis

**Figure 2:**
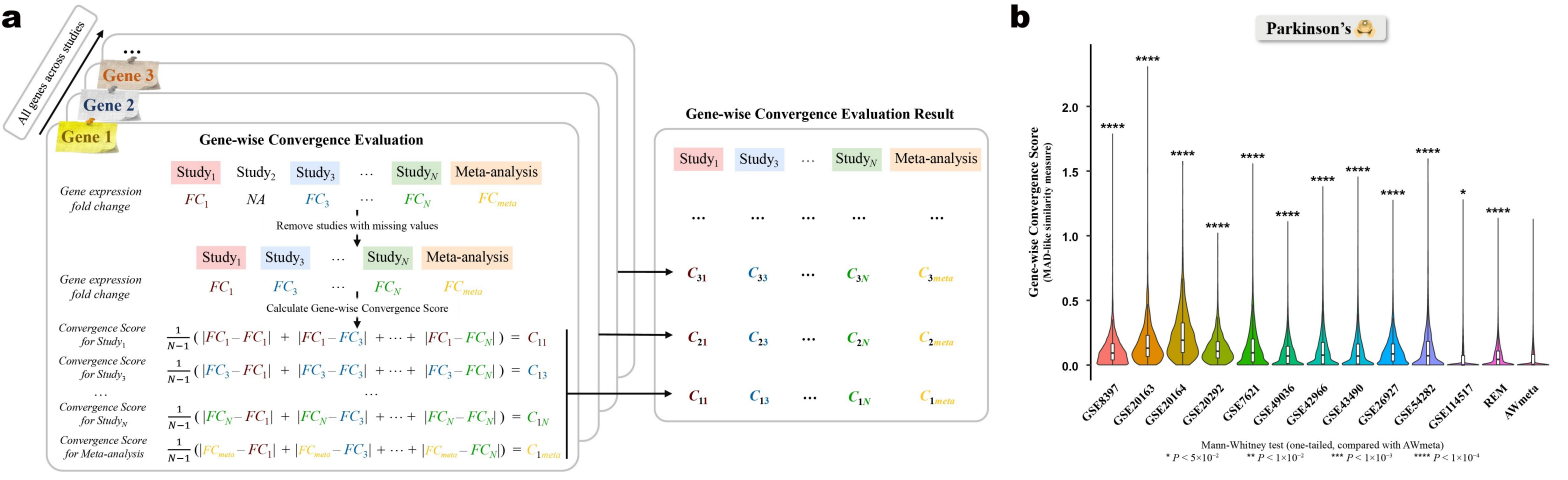

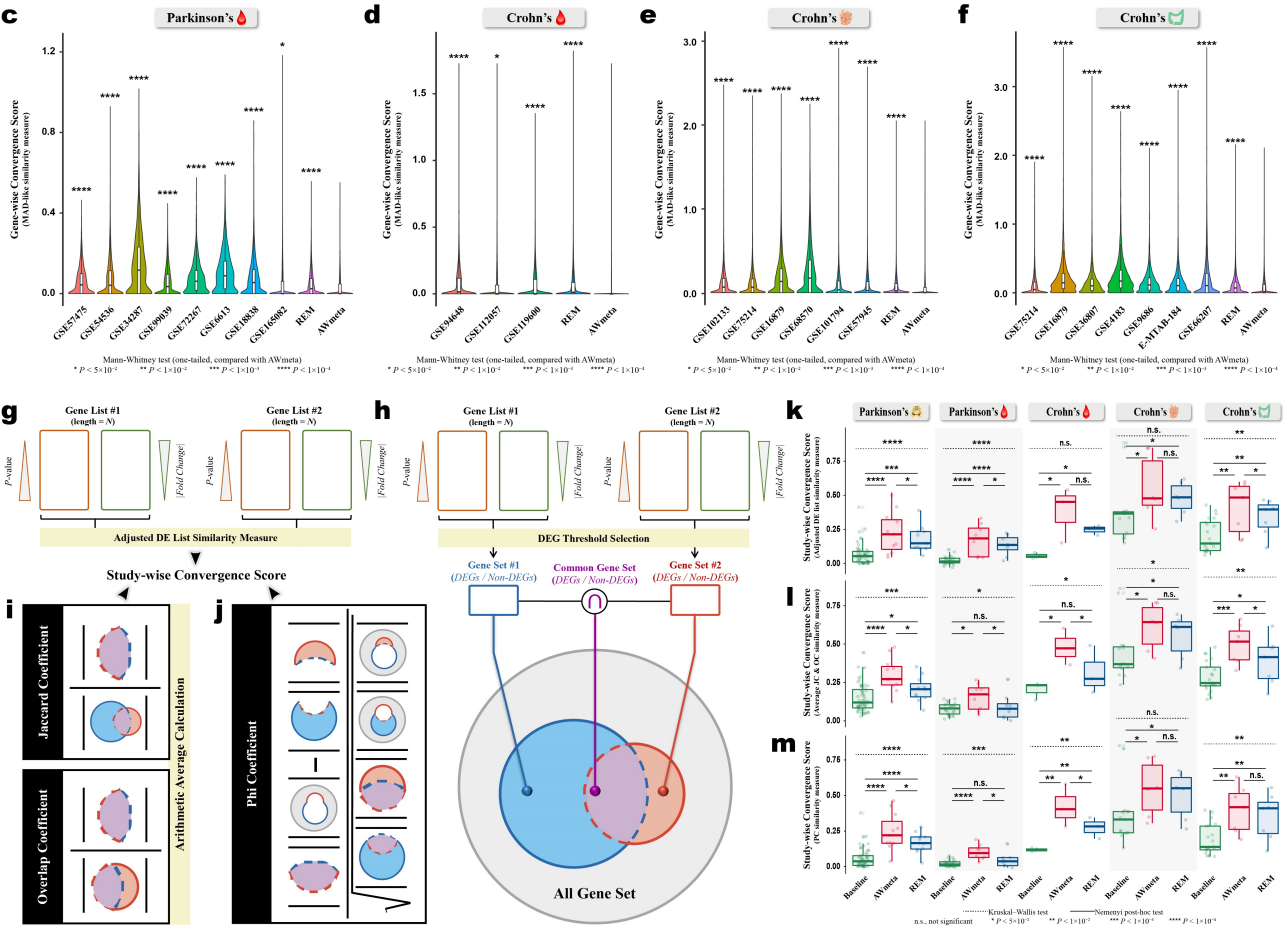
AWmeta establishes superior gene- and study-wise convergence in gene differential expression of Parkinson’s and Crohn’s disease. (**a**) Schematic of the gene-wise convergence evaluation procedure for gene differential expression. Mean absolute deviation (MAD)-like similarity measure was utilized to quantify the per-gene fold change (|log_2_FC|) similarity among AWmeta, REM, and original studies, with a smaller value indicating better convergence. (**b**–**f**) Gene-wise convergence results in five disease tissues. Statistical significance against AWmeta for gene-wise convergence comparisons was determined by a one-tailed Mann–Whitney test. (**g**–**j**) Workflow of study-wise convergence score calculation for gene differential expression by adjusted DE list similarity, the average of Jaccard (JC), overlap coefficient (OC), and phi coefficient (PC). (**k**–**m**) Study-wise convergence assessment results for three similarity measures in five disease tissues with FDR < 0.05 and |log_2_FC| > log_2_1.2. For comparison purposes, results from original studies serve as reference baselines. Overall study-wise convergence differences among AWmeta, REM, and baselines were tested with the Kruskal–Wallis test, followed by the Nemenyi post-hoc test for pairwise comparisons. Boxplot bounds show interquartile ranges (IQR), centers indicate median values, and whiskers extend to 1.5 × IQR. The following icons represent different tissue sources: 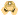
—substantia nigra; 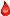
—peripheral blood; 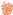
—ileal mucosa; 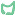
—colonic mucosa. n.s., not significant. *, $p<5\times 10^{-2}$. **, $p<1\times 10^{-2}$. ***, $p<1\times 10^{-3}$. ****, $p<1\times 10^{-4}$.

To assess per-gene differential expression agreement between meta-analysis ($FC_{meta}$) and original constituent studies ($FC_{i}$), a mean absolute deviation (MAD)-like gene-wise convergence score was calculated (Figure 2a). For each gene G,

(7) $$C_{Gmeta}=\frac{1}{|S_{gene}|}\sum_{i\in S_{gene}^{\prime }}\left|FC_{meta}-FC_{i}\right|$$

where $S_{gene}$ and $S_{gene}^{\prime }$ denote the valid study set and corresponding indices for the gene respectively, $|S_{gene}|$ the cardinality of the set $S_{gene}$, $FC_{meta}=M$ from Equation (6), and $FC_{i}$ is the study-exclusive log_2_-based fold change. A lower $C_{Gmeta}$ implies better agreement between the meta-analysis and original study estimates within $S_{gene}$. For baseline comparison, a gene-wise convergence score was also computed for each original Study_*j*_ $\in S_{gene}$:

(8) $$C_{Gj}=\frac{1}{|S_{gene}|}\sum_{i\in S_{gene}^{\prime }}\left|FC_{j}-FC_{i}\right|$$

which represents the MAD of Study_*j*_’s fold change from all other contributing studies. This internal consistency benchmark enables direct contrast of AWmeta’s and REM’s convergence performance against the inherent agreement among the original datasets. All comparisons used a one-tailed Mann–Whitney test against AWmeta.

#### 2.4.4. Study-Wise Convergence Assessment for Gene Differential Expression Meta-Analysis

To rigorously evaluate the consistency between gene lists derived from meta-analysis methods and those from the original studies, three complementary approaches were employed. For all metrics, higher scores indicate superior study-level convergence.

1. Adjusted rankeD genE (DE) list similarity: Our first approach quantifies concordance using a rank-sensitive similarity metric that is critically weighted towards top-ranked genes (Figure 2g). The schematic diagram of this approach can be found in Supplementary Figure S7. To construct robustly ordered gene lists ($G_{\mathrm{meta}}$ for the meta-analysis; $G_{i}$ for Study*_i_*), we first devised a composite rank for each gene by multiplying its *p*-value rank (ascending) with its |log_2_FC| rank (descending), thereby integrating statistical significance and effect size. The weighted similarity $S(G_{meta},G_{i})$ between the meta-analysis and each original study gene lists (containing *N* genes) was computed using a non-linear weighting scheme [36], which emphasizes the top-ranked gene concordance:
  (9) $$S\left(G_{meta},G_{i}\right)=\sum_{n=1}^{N}e^{-\alpha n}O_{n}\left(G_{meta},G_{i}\right)$$
  where $O_{n}\left(G_{meta},G_{i}\right)$ is the number of common genes in the top *n* positions, and α is a weighting exponent (0.001). This score was then normalized to the interval [−1,1] [34] yielding the adjusted similarity:
  (10) $$S_{adj}\left(G_{meta},G_{i}\right)=\frac{S\left(G_{meta},G_{i}\right)-E_{\mathrm{null}}\left(S\left(G_{meta},G_{i}\right)\right)}{\max\left(S\left(G_{meta},G_{i}\right)\right)-E_{\mathrm{null}}\left(S\left(G_{meta},G_{i}\right)\right)}$$
  where $E_{\mathrm{null}}\left(S\left(G_{meta},G_{i}\right)\right)=\sum_{n=1}^{N}\frac{n^{2}}{N}e^{-\alpha n}$ and $\max\left(S\left(G_{meta},G_{i}\right)\right)=\sum_{n=1}^{N}ne^{-\alpha n}$ are the expected and maximum scores under a null hypothesis of random gene lists.
2. Set-based overlap similarity: To circumvent the limitations of the above rank-dependent approach, which is sensitive to gene ranking variations while potentially overlooking consistent differential expression patterns, study-wise convergence was assessed using a set-based overlap metric that exclusively evaluates binary DEG classification concordance (Figure 2h,i). Here, DEG sets were determined for both the meta-analysis ($Set_{meta}$) and individual studies ($Set_{i}$) using predefined statistical thresholds, thereby focusing analytical power on reproducible differential expression status irrespective of positional gene rankings. Two metrics were calculated: Jaccard coefficient (JC) ($JC_{i}=|Set_{meta}\cap Set_{i}|/|Set_{meta}\cup Set_{i}|$) and overlap coefficients (OC) ($OC_{i}=|Set_{meta}\cap Set_{i}|/\min(|Set_{meta}|,|Set_{i}\left|\right)$). The convergence metric for the meta-analysis relative to Study*_i_* was the arithmetic mean $(JC_{i}+OC_{i})/2$.
3. Phi coefficient (PC) similarity: Finally, PC [37] was utilized to measure the association between DEG classifications (Figure 2h,j). This approach considers the extreme case where shared DEGs or non-DEGs between two gene sets might be randomly generated.
  For each comparison between the meta-analysis and an original Study*_i_*, a 2 × 2 contingency table was constructed to categorize all genes as DEG or non-DEG in both datasets, and the corresponding PC ($PC_{i}$) was then calculated as
  (11) $$PC_{i}=\frac{n_{11}n_{22}-n_{12}n_{21}}{\sqrt{n_{1\cdot }n_{2\cdot }n_{\cdot 1}n_{\cdot 2}}}$$
  where $n_{11}$ represents DEGs, $n_{22}$ non-DEGs in both datasets, and $n_{12}$ and $n_{21}$ represent exclusively-classified DEGs for the binary datasets. The row and column sums are denoted by $n_{1\cdot }$, $n_{2\cdot }$, $n_{\cdot 1}$, and $n_{\cdot 2}$.

To establish a performance baseline, all three convergence metrics were computed for every pairwise combination of the original studies. Overall study-wise convergence differences among AWmeta, REM, and baselines were tested by the Kruskal–Wallis test, followed by the Nemenyi post-hoc test for pairwise comparisons.

For the set-based and PC metrics, which rely on binary DEG and non-DEG classification, we note that these outcomes are mutually exclusive and complementary, and therefore report the results derived from the DEG sets for clarity and conciseness.

#### 2.4.5. Stability and Robustness Assessment of Transcriptomic Meta-Analysis

To demonstrate AWmeta’s resilience, both stability against stochastic sampling and robustness against dataset perturbations were evaluated using the adjusted DE list similarity (Section 2.4.4 and Supplementary Figure S7).

**Figure 3:**
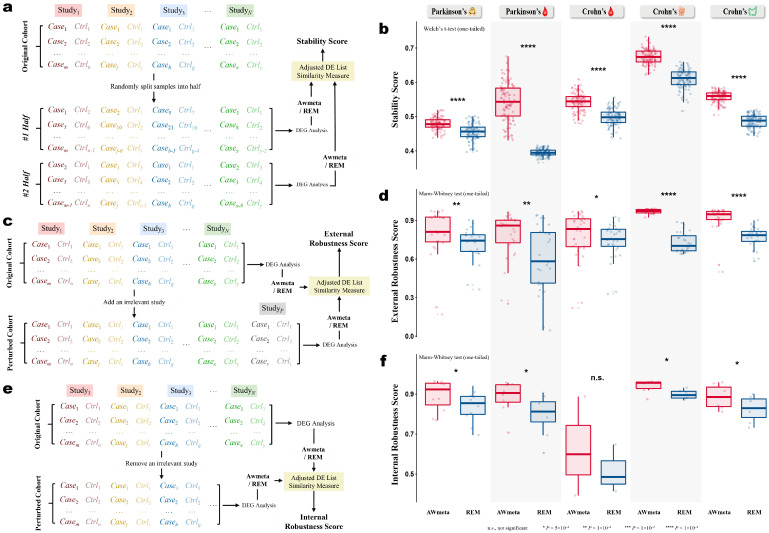
AWmeta delivers remarkable stability and robustness in transcriptomic meta-analysis of Parkinson’s and Crohn’s disease. (**a**) Workflow for evaluating the stability of transcriptomic meta-analysis. (**b**) Stability assessment against AWmeta and REM with one-tailed Welch’s *t*-test. (**c**) Conceptual schematic of external robustness score calculation. (**d**) External robustness assessment results in transcriptomic meta-analysis across five disease tissues, with a one-tailed Mann–Whitney test. (**e**) Conceptual schematic of internal robustness score calculation. (**f**) Internal robustness assessment results in transcriptomic meta-analysis across five disease tissues, with a one-tailed Mann–Whitney test. Boxplot bounds show interquartile ranges (IQR), centers indicate median values, and whiskers extend to 1.5 × IQR. The following icons represent different tissue sources: 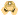
—substantia nigra; 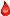
—peripheral blood; 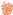
—ileal mucosa; and 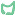
—colonic mucosa. n.s., not significant. *, $p<5\times 10^{-2}$. **, $p<1\times 10^{-2}$. ***, $p<1\times 10^{-3}$. ****, $p<1\times 10^{-4}$.

1. Within-study subsampling stability: For each disease tissue, case and control samples of every constituent study were randomly partitioned into two equal sub-cohorts, yielding paired “half-study” datasets. Each half-study set underwent independent DEG analysis and subsequent meta-analysis. The similarity between the resulting ordered gene lists was computed over 100 bootstrap replicates, quantifying stability under within-study sampling (Figure 3a). AWmeta and REM stability distributions were compared via one-tailed Welch’s *t*-test.
2. External robustness: For each disease tissue, resilience to new data was assessed by sequentially incorporating one external study into the original meta-analysis (Figure 3c). This independent external study cohort consists of all non-target disease tissue studies derived from our full panel of 35 transcriptomic datasets. For each addition, the meta-analysis procedure was implemented before and after study inclusion, and then adjusted DE list similarity between resulting ordered gene lists was computed to measure the impact of disparate external data (AWmeta versus REM, by the one-tailed Mann–Whitney test). This design mimics a real-world scenario where a thematically misaligned study is inadvertently included in a meta-analysis.
3. Internal robustness: Sensitivity to study omission was evaluated by performing leave-one-study-out analyses (Figure 3e): each original study was removed in turn, and meta-analyses were rerun on the reduced datasets. The similarity between each leave-one-study-out and the full-cohort ranked gene lists, across all iterations, quantified internal robustness (AWmeta versus REM, by the one-tailed Mann–Whitney test).

#### 2.4.6. Biological Relevance Assessment of Gene Differential Expression Meta-Analysis

To quantify disease-context relevance of gene differential expression meta-analysis, we assembled benchmark gene sets for Parkinson’s and Crohn’s disease from three sources: (1) DisGeNET [38] with gene-disease association (GDA) score >0.2 [39,40], (2) MalaCards [41], and (3) our disease-related genetic variation corpus [42], manually curated and constructed, from published genetic association studies of Parkinson’s and Crohn’s disease. For reference comparisons, original study-derived biological relevance results serve as baselines.

Since fold change and *p*-value are both essential for screening DEGs, for each method (AWmeta, REM or baselines), all analyzed genes were ranked twice—(i) by descending |log_2_FC|, (ii) by ascending *p*-value—then each benchmark gene’s ranks were multiplied to integrate rank information from fold change and *p*-value:

(12) $$\mathrm{Integrated}\;\mathrm{Rank}={\mathrm{Rank}}_{\left|\log_{2}\mathrm{FC}\right|}\times {\mathrm{Rank}}_{p-\mathrm{value}}$$

**Figure 4:**
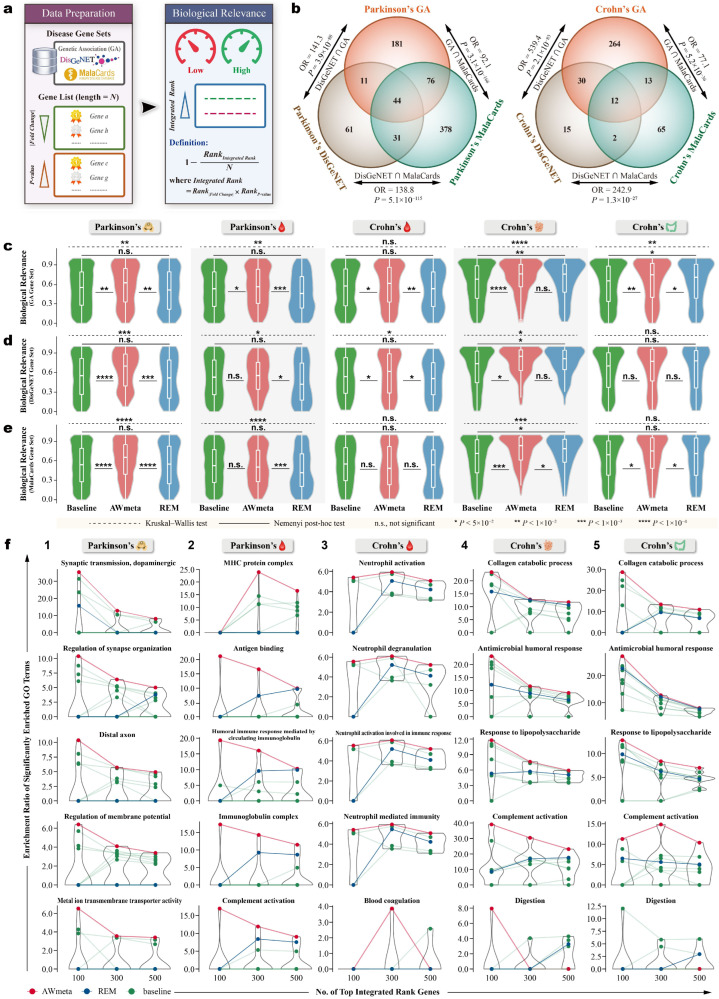
AWmeta enhances identification of and tissue-contextual mechanism interpretation from Parkinson’s and Crohn’s disease genes. (**a**) Workflow for quantifying gene-wise biological relevance against three benchmark gene sets of Parkinson’s and Crohn’s disease from genetic association (GA) variation corpus, DisGeNET, and MalaCards, with higher scores indicating stronger tissue-contextual disease associations. Detailed textual description of this biological relevance evaluation procedure can be referred to in Section 2.4.6. (**b**) Pairwise coherence analysis of the three benchmark gene sets for Parkinson’s and Crohn’s disease. The degree of overlap between benchmarks was quantified using odds ratios (OR), with statistical significance determined by Fisher’s exact test. (**c**–**e**) Biological relevance evaluations on AWmeta (red), REM (blue), and baselines (green) against GA, DisGeNET, and MalaCards benchmarks in five disease tissues, where higher scores (y-axis) denote enhanced biological relevance. Original study-derived biological relevance results serve as reference baselines. Overall differences in biological relevance scores were assessed using the Kruskal–Wallis and Nemenyi post-hoc test for pairwise comparisons (AWmeta versus REM, AWmeta versus baseline, and REM versus baseline). Boxplot bounds indicate interquartile ranges (IQR), centers denote median values, and whiskers extend to 1.5 × IQR. (**f**) Representative GO enrichment trajectories in five disease tissues across top integrated rank (incl. top 100, 300, and 500) genes identified by AWmeta (red), REM (blue), and baselines (green), with higher enrichment ratio (y-axis) indicating stronger disease-tissue involvement. Original study-derived enrichments serve as baselines. Connected lines of enrichment ratio for a significant GO term visualize the density distribution of GO-term-relevant genes across various integrated rank thresholds (x-axis), where curve peaks indicate integrated ranks at which these genes are most enriched. The following icons represent different tissue sources: 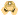
—substantia nigra; 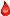
—peripheral blood; 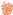
—ileal mucosa; 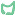
—colonic mucosa. n.s., not significant. *, $p<5\times 10^{-2}$. **, $p<1\times 10^{-2}$. ***, $p<1\times 10^{-3}$. ****, $p<1\times 10^{-4}$.

Benchmark genes were then re-ranked according to this IntegratedRank (ascending) to obtain ${\mathrm{Rank}}_{\mathrm{Integrated}\;\mathrm{Rank}}$. The biological relevance score was calculated for each benchmark gene as

(13) $$\mathrm{Biological}\;\mathrm{Relevance}=1-\frac{{\mathrm{Rank}}_{\mathrm{Integrated}\;\mathrm{Rank}}}{N}$$

where *N* is the size of the gene list from AWmeta, REM, or baselines. Higher scores reflect greater biological relevance, signifying that benchmark genes attain superior rankings through combining statistical significance and fold change (Figure 4a). This rank-based score accounts for gene list size heterogeneity and avoids arbitrary DEG thresholds. Biological relevance distributions from AWmeta, REM, and baselines were compared using Kruskal-Wallis and Nemenyi post-hoc tests.

#### 2.4.7. Gene Ontology (GO) Enrichment Analysis of Gene Differential Expression Meta-Analysis

To explore tissue-wise Parkinson’s and Crohn’s disease mechanisms hidden behind meta-analysis prioritized genes, GO enrichment was further implemented by over-representation analysis with clusterProfiler [43]. To avoid arbitrariness, three thresholds (100, 300, and 500) were used to select the number of top integrated rank genes (Section 2.4.6 and Figure 4a). The enrichment ratio quantifies the degree to which GO terms are significantly enriched in relevant disease tissues:

(14) $$\mathrm{Enrichment}\;\mathrm{ratio}=\frac{\mathrm{Gene}\;\mathrm{ratio}}{\mathrm{Background}\;\mathrm{ratio}}$$

where gene ratio is the proportion of genes annotated to a specific GO term within the top integrated rank genes, and background ratio represents the analogous fraction across the whole human genes with GO annotations. GO terms with higher enrichment ratios are more likely to be involved in a given disease tissue. GO terms with multiple-testing-adjusted *p*-values < 0.05 were subjected to enrichment ratio calculation. For comparison, GO enrichments derived from original studies served as baselines.

## 3. Results

### 3.1. AWmeta Secures Consistent Higher-Fidelity DEG Identification Across Transcriptomic Contexts of Parkinson’s and Crohn’s Disease

The primary goal of transcriptomic meta-analysis is to enhance statistical power for identifying DEGs reliably, i.e., to detect more subtle yet vital DEGs, typically defined by statistical significance and fold-change thresholds (Figure 1b). Systematically benchmarking across five distinct disease tissue contexts using nine combinations of statistical significance (0.01, 0.05, and 0.10) and fold-change thresholds ($\log_{2}1.2$, $\log_{2}1.5$, and $\log_{2}2.0$), AWmeta consistently identified significantly more DEGs than REM (*p* < $10^{-4}$, one-tailed Welch’s *t*-test over 100 bootstrap iterations; Figure 1c and Supplementary Figure S2). For instance, under a specific threshold combination (*p* < 0.01 and $|\log_{2}\mathrm{FC}|>\log_{2}1.2$), AWmeta yielded 69–475% increases in detected DEGs versus REM across all tissues (Figure 1c), which demonstrates AWmeta’s superior sensitivity in DEG detection.

A key challenge in meta-analysis is to increase statistical power while rigorously controlling false positives. To formally evaluate this trade-off, a semi-synthetic simulation framework was designed to assess DEG discrimination (Section 2.4.2). Within this framework, the performance of AWmeta was systematically challenged by degrading the biological signal in a controlled manner, achieved by permuting sample labels in a minimum, median, and maximum allowable number of studies within each tissue context (Figure 1d–f). This perturbation design enabled a rigorous assessment of AWmeta’s resilience across diverse data quality landscapes, a critical feature for real-world applications.

Across all simulated noise levels, AWmeta consistently outperformed or was comparable to REM in DEG discrimination. Under minimum-permuted low-noise condition, AWmeta demonstrated a clear and significant advantage across nearly all tissue contexts and DEG thresholds (*p* < $10^{-4},10^{-3},10^{-2}$ or 0.05, one-tailed Mann–Whitney test; Figure 1g and Supplementary Figure S3). As expected, performance decayed for both methods with increasing noise from median and maximum study permutations. However, AWmeta’s superiority over REM was not only maintained but often became more pronounced under these more challenging conditions (*p* < $10^{-4}$, one-tailed Mann–Whitney test; Figure 1h,i and Supplementary Figures S4 and S5). Notably, AWmeta’s performance remained remarkably robust even in high-noise scenarios, with median AUROC and AUPRC exceeding 0.85 in most cases (Supplementary Figure S5), highlighting its ability to effectively discount noise from potentially confounding studies. Taken together, these results demonstrate that AWmeta achieves a superior balance between heightened sensitivity and robust discrimination for higher-fidelity DEG identification from heterogeneous transcriptomic datasets.

### 3.2. AWmeta Establishes Superior Gene- and Study-Wise Convergence in Gene Differential Expression of Parkinson’s and Crohn’s Disease

To rigorously assess AWmeta’s ability to synthesize a consensus biological signal from heterogeneous transcriptomic datasets, gene differential expression convergence at both gene and study levels was evaluated. Gene-wise convergence, i.e., the proximity of a gene’s meta effect-size estimates to those from original studies, was first quantified using MAD between meta and original fold changes (Figure 2a), where lower MADs signify more accurate biological representations.

AWmeta consistently yielded significantly lower gene-wise convergence scores in all five disease tissues compared to both REM and baseline ones (Figure 2b–f; $p<10^{-4}$ or 0.05, one-tailed Mann–Whitney test against AWmeta). Notably, while some original studies occasionally outperformed REM in specific contexts, AWmeta (merely 57–74% of REM) consistently achieved lower scores than any original study across all tissues, which suggests its capacity to robustly identify and integrate reliable signals while effectively down-weighting divergent studies, thereby providing a superior consensus representation of the gene expression landscape. Since DEGs are more likely involved in disease processes than non-DEGs, we further confirmed that this superior performance was still evident for these specific genes by the same assessment paradigm using nine distinct thresholds, combining three significance levels (0.01, 0.05, and 0.10) and three fold-change cutoffs ($\log_{2}1.2$, $\log_{2}1.5$, and $\log_{2}2.0$). Across all threshold and disease tissue scenarios, AWmeta maintained evidently lower convergence scores, accounting for 56–80% of REM (Supplementary Figure S6a–e; $p<10^{-4},10^{-3},10^{-2}$ or 0.05, one-tailed Mann–Whitney test against AWmeta), which underscores AWmeta’s effectiveness for deriving robust fold-change estimates for disease-relevant genes, independent of specific statistical criteria.

Next, we evaluated study-wise convergence to determine how well the meta-analytic results reflect the collective evidence across all contributing studies. Three complementary approaches were employed: an adjusted rank-sensitive similarity metric emphasizing top-ranked genes (denoted “adjusted DE list similarity” thereafter), the arithmetic mean of JC and OC for DEG concordance, and PC to assess classification agreement beyond chance (Figure 2g–j, Supplementary Figure S7). Higher scores indicate better study-wise convergence for all metrics.

Across the five disease tissues, AWmeta consistently achieved significantly higher study-wise convergence scores than baselines representing original inter-study agreement, with dramatic 30–1166% improvements (Figure 2k–m; *p* < 0.05 and $|\log_{2}\mathrm{FC}|>\log_{2}1.2$ where applicable). While overall convergence scores tended to be lower in Parkinson’s over Crohn’s disease tissues, potentially reflecting higher inherent variability within these specific disease contexts, AWmeta significantly outperformed REM in the majority (10 out of 15) of comparisons across different metrics and tissues, performing comparably otherwise, particularly pronounced in tissues like Parkinson’s and Crohn’s peripheral blood, where AWmeta’s convergence scores improved by 35–156% compared to REM (Figure 2k–m). To validate that these findings for the JC/OC and PC metrics are not artifacts of a specific threshold, we further performed corresponding study-wise convergence evaluations across nine different DEG cutoffs and found AWmeta’s superior performance DEG-cutoff-independent (Supplementary Figure S8). These results indicate that the gene differential expression results processed by AWmeta are more representative of the faithful consensus signal across studies than those derived from REM or original studies.

### 3.3. AWmeta Delivers Remarkable Stability and Robustness in Transcriptomic Meta-Analysis of Parkinson’s and Crohn’s Disease

We sought to determine whether AWmeta’s adaptively weighted strategy confers superior stability and robustness to gene differential expression meta-estimates against REM and designed quantitative metrics to evaluate consistency over random splits and resilience to systematic perturbations across the five disease tissues.

First, stability was assessed by quantifying the concordance of ranked gene differential lists derived from randomly halved sample sets within each study, a process replicated across 100 iterations (Figure 3a). Across all five disease tissues, AWmeta exhibited markedly higher stability scores relative to REM (Figure 3b; *p* < $10^{-4}$, one-tailed Welch’s *t*-test), underscoring its enhanced consistency under data rationing. The observation that median stability scores for both methods were below 0.7 is likely attributable to the inherently reduced statistical power and study-wise convergence that accompanies halving the sample size.

We then challenged the robustness of each method against two distinct forms of perturbation: external interference, simulated by the inclusion of a thematically unrelated study (Figure 3c), and internal fragility, evaluated through a systematic leave-one-study-out procedure (Figure 3e). Against external interference, AWmeta displayed remarkable resilience with median robustness scores above 0.8 and established a significant performance margin over REM across all tissues (Figure 3d; *p* < $10^{-4},10^{-2}$ or 0.05, one-tailed Mann–Whitney test). This capacity to resist discordant data is a direct consequence of AWmeta’s adaptive weighting scheme, which effectively minimizes the influence of outlier studies. In the internal robustness assessment, AWmeta again achieved significantly higher scores than REM in four of the five tissues (Figure 3f; *p* < 0.05, one-tailed Mann–Whitney test). The sole exception was Crohn’s peripheral blood, where the small cohort of only three studies constrained the median robustness scores to fall below 0.6 for both methods. Notably, in tissues comprising six or more studies, AWmeta achieved exceptional median internal robustness scores around 0.9, demonstrating highly consistent results even upon the exclusion of individual constituent studies.

These rigorous stress tests validate that AWmeta’s adaptive weighting architecture endows the meta-analytic process with significantly strengthened stability and robustness. This reinforcement ensures the derivation of more dependable biological insights when integrating diverse and inherently heterogeneous transcriptomic datasets.

### 3.4. AWmeta Facilitates Prioritization of Parkinson’s and Crohn’s Disease Genes

A pivotal determinant of a meta-analysis method’s utility is its capacity to prioritize genes of genuine pathological importance. To rigorously assess this, the biological relevance of gene rankings from AWmeta, REM, and the original studies (as baselines) was quantified against authoritative Parkinson’s and Crohn’s disease-gene benchmarks—compiled from DisGeNET, MalaCards, and a well-curated genetic variation corpus—using a custom metric that integrates both statistical significance and fold-change magnitude, which provides an objective and threshold-agnostic evaluation of gene prioritization performance (Figure 4a).

Prior to assessing performance, we first validated the coherence of our benchmark gene sets, with overlap magnitude quantified using odds ratio (OR) and statistical significance determined by Fisher’s exact test. Pairwise comparisons revealed substantial overlaps among the three independent sources for both Parkinson’s disease (e.g., DisGeNET versus MalaCards, OR =138.8, $p=5.1\times 10^{-115}$) and Crohn’s disease (e.g., DisGeNET versus MalaCards, OR =242.9, $p=1.3\times 10^{-27}$) (Figure 4b). This strong reciprocal consistency affirmed their utility for a reliable evaluation of biological relevance.

Our primary analysis revealed that AWmeta consistently generates more biologically meaningful gene rankings than REM and the baseline studies (*p* < $10^{-4},10^{-3},10^{-2}$ or 0.05, Nemenyi post-hoc test; Figure 4c–e). Specifically, when benchmarked against our genetic variant corpus, AWmeta achieved significantly higher relevance scores across all interrogated tissues (Figure 4c). This superior performance extended to the DisGeNET benchmark in critical disease tissues, including Parkinson’s substantia nigra and Crohn’s peripheral blood and ileal mucosa (Figure 4d). A similar advantage was observed using the MalaCards benchmark for Parkinson’s substantia nigra and Crohn’s ileal and colonic mucosa (Figure 4e). Notably, AWmeta’s superiority was particularly pronounced in the primary disease-affected tissues—Parkinson’s substantia nigra and Crohn’s ileal mucosa—where it surpassed baselines across all three independent benchmarks. Cumulatively, in 11 instances of the 15 tissue-benchmark comparisons (5 tissues × 3 benchmarks), AWmeta’s scores were significantly higher than those of both the baselines and REM. In contrast, REM failed to offer a significant improvement over baseline scores in 11 of 15 comparisons (*p* > 0.05, Nemenyi post-hoc test; Figure 4c–e), underscoring its limited ability to distill more disease-associated genes through summarizing original transcriptomic datasets.

These results establish that AWmeta’s gene prioritization is not merely a statistical refinement but contributes to detecting more genes of pathological relevance in various tissues of Parkinson’s and Crohn’s disease. By more effectively elevating established disease-associated genes to the top of integrated rankings, AWmeta paves the way for providing a clearer and more accurate representation of the underlying tissue-level pathologies in Parkinson’s and Crohn’s disease.

### 3.5. AWmeta Enhances Tissue-Contextual Mechanism Interpretation from Prioritized Parkinson’s and Crohn’s Disease Genes

GO enrichment was further carried out to dissect tissue-wise Parkinson’s and Crohn’s disease mechanisms hidden behind meta-analysis-prioritized genes. For clarity, only a few representative enriched GO terms are used for subsequent interpretation of disease mechanisms, and the complete GO enrichment results are provided in our GitHub repository (https://github.com/YanshiHu/AWmeta, accessed on 14 May 2026).

Compared with REM and baselines, representative GO terms enriched in AWmeta-derived top integrated rank genes consistently exhibited the highest enrichment ratios across nearly all five disease tissues (Figure 4f), demonstrating AWmeta’s enhanced capacity for disease-relevant gene prioritization in tissue-specific contexts; in contrast, REM underperformed relative to some baselines, reflecting diminished biological relevance within its gene sets. For instance, biological processes related to synaptic organization and transmission (“synaptic transmission, dopaminergic”, “regulation of synapse organization”, and “distal axon”) were significantly enriched in Parkinson’s substantia nigra (Figure 4f(1)), consistent with their known involvement in Parkinson’s pathogenesis [44,45]. Likewise, "metal ion transmembrane transporter activity" and "regulation of membrane potential" were significantly enriched (Figure 4f(1)), highlighting their pivotal roles in Parkinson’s substantia nigra-involved mechanisms [46,47,48]. AWmeta achieved the highest enrichment ratio for these GO terms, a trend robust across all prioritized gene selection thresholds (100/300/500). In the context of Parkinson’s substantia nigra, the enrichment ratios of representative GO terms for AWmeta and some baselines monotonically decreased with more integrated rank genes included, suggesting that these term-related genes are concentrated at the very top of the ranked lists; REM-prioritized genes exhibited delayed or absent enrichment across all five representative GO terms, further underscoring its impaired capacity to capture these contextual biological functions of Parkinson’s substantia nigra (Figure 4f(1)).

Given the well-established inflammatory pathogenesis of Parkinson’s and Crohn’s disease in non-hematopoietic tissues [49,50], we hypothesized that blood-borne gene signatures would reflect systemic immune dysregulation and vascular barrier impairment at disease-relevant interfaces: Parkinson’s blood-brain barrier and Crohn’s intestinal vasculature. Peripheral blood analyses for Parkinson’s disease revealed significant enrichment of immune-related GO terms, including “MHC protein complex” [51], “antigen binding” [52], and “immunoglobulin complex”, with AWmeta reaching the highest enrichment ratio (Figure 4f(2)). The circulatory specificity was further evidenced by “humoral immune response mediated by circulating immunoglobulin”. Similarly, in Crohn’s peripheral blood, significant neutrophil-related GO terms, incl. “neutrophil degranulation", “neutrophil activation involved in immune response", “neutrophil mediated immunity” and “neutrophil activation”, with AWmeta as top performer (Figure 4f(3)), indicated the involvement of immune-inflammatory processes [53]. Furthermore, significant Parkinson’s “complement activation” and Crohn’s “blood coagulation” provided disease-specific vascular insights (Figure 4f(2,3)). Aberrant complement system activity may imply blood-brain barrier disruption in Parkinson’s patients [54], whereas increased venous thromboembolism risk in Crohn’s patients due to abnormal coagulation [55] indicates intestinal vascular barrier impairment [56]. It is noteworthy that enrichment ratios of GO term “MHC protein complex” in Parkinson’s blood (Figure 4f(2)) and all five illustrative GO terms in Crohn’s blood (Figure 4f(3)) peak within the top 300 integrated rank genes, which suggests most genes associated with these GO terms fall within the integrated ranking range of 100 to 300. Notably, two GO terms exhibit an even more extreme pattern: “MHC protein complex” in Parkinson’s blood and “blood coagulation” in Crohn’s blood show an enrichment ratio of exactly zero within the top 100 ranked genes, implying no genes annotated to these two GO terms appear in the top 100 of the integrated ranking.

Functional enrichment experiments in ileal and colonic mucosa of Crohn’s disease illustrated “collagen catabolic process” was most pronounced by AWmeta (Figure 4f(4,5)). This biological process has been well-documented to play a critical role in extracellular matrix remodeling of ileal and colonic mucosa [57], which represent primary pathological sites in Crohn’s disease [50]. At the same time, significantly enriched GO terms related to gut microbiota dysbiosis, such as “antimicrobial humoral response” and “response to lipopolysaccharide” (Figure 4f(4,5)), further aligned with Crohn’s pathogenesis [58,59].

While Crohn’s ileal and colonic mucosa share multifaceted similarities, the AWmeta-prioritized genes can be used to reveal two distinct functional dichotomies: complement activation and digestive function. Both mucosal compartments were characterized by detectable the GO term “complement activation”, yet the corresponding enrichment ratios in the ileal mucosa were 2–4-fold higher than those in the colonic mucosa (Figure 4f(4,5)). This tissue-specific disparity is biologically supported by immunofluorescence staining and single-cell transcriptomic evidence of more prominent complement activation within ileal mucosa [60,61]. Furthermore, our functional enrichment analysis of AWmeta-derived genes uncovered a clear tissue partition in digestive roles: “digestion” was specifically enriched in ileal mucosa rather than colonic mucosa. Specifically, AWmeta exclusively detected “digestion” within the top 100 ileal mucosa genes with the highest enrichment ratio, whereas REM and baselines showed delayed, lower enrichment in the top 300/500 genes (Figure 4f(4)); conversely, AWmeta consistently excluded “digestion” from colonic mucosa enrichments, with its enrichment ratio constantly being zero in all three integrated rank cutoffs, in contrast to sporadic false positives by these counterparts (Figure 4f(5)). This finding is evidenced by established biological knowledge that the ileal mucosa uniquely mediates hydrolase-driven enzymatic digestion, whereas the colonic mucosa plays no substantive role in chemical digestion [62]. These functional stratifications collectively demonstrate AWmeta’s enhanced pathobiological fidelity in resolving tissue-contextual mechanisms in Crohn’s ileal and colonic mucosa from corresponding prioritized genes.

## 4. Discussion

Transcriptomic meta-analysis is pivotal for distilling robust biological insights from heterogeneous gene expression studies; yet, existing frameworks remain confined to either *p*-value combination or effect-size integration, imposing a trade-off between statistical sensitivity and quantitative fidelity. AWmeta represents the first successful integration of *p*-value and effect-size aggregation methodologies in the transcriptomic meta-analysis field. The core innovation—a cross-module information transfer where optimized weights from *p*-value calculations directly enhance effect size estimation—effectively addresses between-study heterogeneity while maximizing consistent biological signal extraction. Indeed, the substantial variability often observed between studies, visually apparent in metrics like gene-wise convergence (Figure 2b–f with per-study skewed distributions), highlights the prevalence of such heterogeneity and strongly supports the use of random-effects-like frameworks such as AWmeta and REM over simpler fixed-effects models [63]. In our comprehensive evaluation across 35 datasets from Parkinson’s and Crohn’s disease, AWmeta demonstrated superior high-fidelity DEG detection that remained robust under substantial experimental noise (Figure 1c,g–i and Supplementary Figures S2–S5). This enhanced discrimination capacity enabled the identification of subtle yet biologically meaningful expression changes that conventional methods frequently omit, substantially improving the reliability and reproducibility of transcriptomic discoveries.

Our convergence metrics revealed AWmeta’s practical advantages in approximating theoretical true values at both gene and study levels. It is noteworthy that in our gene-wise convergence assessments, some original studies occasionally outperformed standard REM, even without larger sample sizes (Figure 2b–f and Supplementary Figure S6). While not conclusive, this hints that inherent study quality or specific experimental contexts might significantly influence reliability, perhaps as much as sample size itself. REM indiscriminately integrates all studies using inverse-variance weights correlated with sample size, and this can, to some extent, distort the reliability of the consensus estimate, sometimes producing results inferior even to a single moderate-sized but high-quality study. In contrast, AWmeta consistently outperformed both REM and all individual studies in convergence. This suggests that AWmeta’s adaptive weights are not simply tracking sample size, but are successfully identifying and incorporating smaller yet biologically informative studies that REM’s undifferentiated weighting scheme fails to adequately leverage.

The superior biological relevance of AWmeta’s findings was rigorously established through two orthogonal and complementary assessment paradigms: (1) Using authoritative disease-specific gene sets from DisGeNET, MalaCards, and an in-house genetic variant corpus, AWmeta demonstrated significantly enhanced biological meaningfulness. In 11 of 15 tissue-benchmark combinations, AWmeta outperformed both REM and original studies in biological relevance scoring (Figure 4c–e). This consistent advantage provides researchers with more accurate representations of core disease pathways and creates unprecedented opportunities for discovering novel pathophysiological relationships that remain obscured in conventional analyses. (2) Longitudinal tracking of GO term enrichment across gene rank thresholds revealed AWmeta’s unique capacity to concentrate functionally critical genes within leading ranks. While terms of secondary importance (e.g., “MHC protein complex”, “blood coagulation”) showed delayed enrichment beyond top 300 ranks across all methods (Figure 4f), pathologically central functions exhibited exclusive early enrichment in AWmeta. Crucially, terms like "digestion" in Crohn’s ileal mucosa reached peak enrichment exclusively within AWmeta’s top 100 genes (Figure 4f), with no detection at expanded thresholds; REM failed to detect this pivotal function at both the top 100 and 300 thresholds, achieving only marginal detection at the top 500 (Figure 4f)—demonstrating its fundamental limitations in biological resolution. This enrichment trajectory analysis establishes a dual-purpose paradigm for quantitatively evaluating gene prioritization performance and objectively stratifying biological mechanisms by pathological centrality. Together, these orthogonal validation strategies—leveraging curated knowledgebases and functional enrichment dynamics—provide compelling evidence that AWmeta uniquely reconciles statistical rigor with biological fidelity, transforming heterogeneous transcriptomic data into precisely stratified mechanistic insights.

Several limitations of the current study should be acknowledged. First, our benchmarking is restricted to Parkinson’s disease and Crohn’s disease across five tissue contexts. While this provides a diverse testbed, broader validation across additional diseases and independent external cohorts is needed to establish the generalizability of AWmeta’s advantages. Second, regarding the choice of comparator, our evaluation focuses on REM as the leading effect-size-based method, which is necessary because our core evaluation framework requires fold change estimates that *p*-value-only and rank-based methods cannot provide. A broader comparative evaluation against a wider panel of transcriptomic meta-analysis methods represents an important direction for future work. Third, while the weight optimization in AWmeta is theoretically well-characterized by the original AW-Fisher framework [10], a systematic sensitivity analysis of weight distributions under systematically varied dataset compositions would further illuminate its empirical behavior and is a worthwhile area for future investigation. Fourth, fold change, used as the effect-size estimator in this study, can be sensitive to noise in low-expression genes and does not explicitly model variance in the way that Cohen’s *d* or Hedges’ *g* does. Our reliance on DESeq2’s and limma’s moderated fold change estimates mitigates, but does not eliminate, this sensitivity. Designing a more sophisticated shrinkage-based or variance-standardized effect size estimator for transcriptomic meta-analysis is an important direction for future work. Fifth, although the AW-Fisher module performs a combinatorial search over all possible study subsets, its computational complexity has been reduced from $O(2^{K})$ to O(Klog(K)) for *K* studies [64]. For the current datasets (K≤15), this is readily tractable; however, scalability to meta-analyses with substantially more studies may require heuristic or approximation strategies. Sixth, while our external robustness test demonstrates resilience to the inclusion of a thematically unrelated study, we acknowledge that this is a simplified model for the complexity of real-world batch effects or cross-platform variability. The primary defense against such technical variability lies in the careful, harmonized preprocessing of the input data, and a full characterization of AWmeta’s performance under systematically varied technical batch effects is an important area for future work. Seventh, the current framework is designed for bulk transcriptomic data. Extension to other omics types, such as proteomics, methylomics, or single-cell transcriptomics, is conceptually feasible but would require careful adaptation of the input statistics and variance modeling. Finally, the biological validation relies on curated gene-disease datasets and functional enrichment, which inherently favor well-characterized genes. Notably, novel candidates not present in any benchmark dataset but co-enriched with gold-standard disease genes within the same significantly enriched GO terms may be considered more plausible disease-relevant candidates, providing a computational plausibility check. Nevertheless, experimental validation of these and other novel candidates identified by AWmeta remains essential. Despite these limitations, we believe AWmeta provides a robust and versatile foundation for transcriptomic meta-analysis, and addressing the constraints outlined above represents a clear roadmap for future development.

## 5. Conclusions

In this study, we present AWmeta, a novel transcriptomic meta-analysis approach that integrates *p*-value aggregation and effect-size estimation through an adaptive weighting scheme. Comprehensive evaluation across 35 datasets from Parkinson’s and Crohn’s disease demonstrates that AWmeta achieves higher-fidelity DEG detection with robustly controlled false positives, superior gene- and study-wise convergence in effect-size quantification, and remarkable stability and robustness against external and internal perturbations. AWmeta also prioritizes genes with enhanced biological relevance and enables tissue-contextual interpretation of Parkinson’s and Crohn’s disease mechanisms with improved functional coherence.

These results establish AWmeta as a powerful and robust approach for extracting reliable DEGs from complex and heterogeneous transcriptomic data. By enhancing the reproducibility and biological interpretability of meta-analytic findings, AWmeta provides a valuable tool for expediting the translation of transcriptomic discoveries into biological insights.

## Data Availability

The data supporting the findings of this study are available on GitHub at https://github.com/YanshiHu/AWmeta (accessed on 14 May 2026) or from the corresponding author upon reasonable request.

## Supplementary Materials

The following supporting information can be downloaded at https://www.mdpi.com/article/10.3390/cimb48050530/s1.

## Abbreviations

The following abbreviations are used in this manuscript:

REM	random-effects model
DEG	differentially expressed gene
RNA-seq	RNA-sequencing
FC	fold change
AUROC	area under the receiver operating characteristic curve
AUPRC	area under the precision–recall curve
MAD	mean absolute deviation
IQR	interquartile ranges
JC	Jaccard coefficient
OC	overlap coefficient
PC	phi coefficient
OR	odds ratio
GO	Gene Ontology
GA	genetic association
