# Supplementary material for: AWmeta Empowers Adaptively Weighted Transcriptomic Meta-Analysis
Source: Curr Issues Mol Biol. 2026 May 19;48(5):530. doi: 10.3390/cimb48050530 (PMC13205005; doi:10.3390/cimb48050530)
Supplement: Supplementary file 1 [file cimb-48-00530-s001.zip › cimb-4286777-supplementary.pdf]

Dataset	Sequencing platform	Dataset source	Tissue source	Samples		
Data IDs	Microarray or next-generation sequencing platforms	GEO, SRA and ArrayExpress	Blood, SN, IM and CM	No. of patients versus normals		
**PD datasets**						
GSE57475	Illumina HumanHT-12 V3.0 expression beadchip	GEO	Peripheral blood	93	versus	49
GSE54536	Illumina HumanHT-12 V4.0 expression beadchip	GEO	Peripheral blood	4	versus	4
GSE34287	ExonHit Human Genome Wide SpliceArray 1.0	GEO	Peripheral blood	19	versus	12
GSE99039	Affymetrix Human Genome U133 Plus 2.0 Array	GEO	Peripheral blood	205	versus	233
GSE72267	Affymetrix Human Genome U133A 2.0 Array	GEO	Peripheral blood	40	versus	19
GSE6613	Affymetrix Human Genome U133A Array	GEO	Peripheral blood	50	versus	22
GSE18838	Affymetrix Human Exon 1.0 ST Array	GEO	Peripheral blood	17	versus	11
GSE165082	Illumina HiSeq 2000	GEO, SRA	Peripheral blood	12	versus	14
GSE114517	Illumina NextSeq 500	GEO, SRA	Substantia nigra	17	versus	12
GSE8397	Affymetrix Human Genome U133A Array	GEO	Substantia nigra	24	versus	13
GSE20163	Affymetrix Human Genome U133A Array	GEO	Substantia nigra	8	versus	9
GSE20164	Affymetrix Human Genome U133A Array	GEO	Substantia nigra	6	versus	5
GSE20292	Affymetrix Human Genome U133A Array	GEO	Substantia nigra	11	versus	15
GSE7621	Affymetrix Human Genome U133 Plus 2.0 Array	GEO	Substantia nigra	16	versus	9
GSE49036	Affymetrix Human Genome U133 Plus 2.0 Array	GEO	Substantia nigra	8	versus	8
GSE42966	Agilent-014850 Whole Human Genome Microarray 4x44K G4112F	GEO	Substantia nigra	9	versus	6
GSE43490	Agilent-014850 Whole Human Genome Microarray 4x44K G4112F	GEO	Substantia nigra	8	versus	5
GSE26927	Illumina humanRef-8 v2.0 expression beadchip	GEO	Substantia nigra	12	versus	8
GSE54282	Affymetrix Human Gene 1.0 ST Array	GEO	Substantia nigra	3	versus	3
**CD datasets**						
GSE119600	Illumina HumanHT-12 V4.0 expression beadchip	GEO	Peripheral blood	95	versus	47
GSE112057	Illumina HiSeq 2000	GEO, SRA	Peripheral blood	60	versus	12
GSE94648	Affymetrix Human Genome U133 Plus 2.0 Array	GEO	Peripheral blood	50	versus	22
GSE102133	Affymetrix Human Gene 1.0 ST Array	GEO	Ileal mucosa	65	versus	12
GSE75214	Affymetrix Human Gene 1.0 ST Array	GEO	Ileal mucosa	51	versus	11
GSE16879	Affymetrix Human Genome U133 Plus 2.0 Array	GEO	Ileal mucosa	18	versus	6
GSE68570	Illumina HumanHT-12 V4.0 expression beadchip	GEO	Ileal mucosa	6	versus	5
GSE101794	Illumina HiSeq 2000	GEO, SRA	Ileal mucosa	198	versus	50
GSE57945	Illumina HiSeq 2000	GEO, SRA	Ileal mucosa	163	versus	42
GSE75214	Affymetrix Human Gene 1.0 ST Array	GEO	Colonic mucosa	8	versus	11
GSE16879	Affymetrix Human Genome U133 Plus 2.0 Array	GEO	Colonic mucosa	19	versus	6
GSE36807	Affymetrix Human Genome U133 Plus 2.0 Array	GEO	Colonic mucosa	13	versus	7
GSE4183	Affymetrix Human Genome U133 Plus 2.0 Array	GEO	Colonic mucosa	5	versus	8
GSE9686	Affymetrix Human Genome U133 Plus 2.0 Array	GEO	Colonic mucosa	11	versus	8
E-MTAB-184	Illumina HumanHT-12 v3.0 expression beadChip	ArrayExpress	Colonic mucosa	15	versus	32
GSE66207	Illumina HiSeq 2500	GEO, SRA	Colonic mucosa	20	versus	13

No. of Parkinson's (PD) / Crohn's disease (CD) patients: 4, 8, 16, 32, 64, 128, 256

No. of normals: 4, 8, 16, 32, 64, 128, 256

GEO · SRA · ArrayExpress · Peripheral blood · Substantia nigra (SN) · Ileal mucosa (IM) · Colonic mucosa (CM) · No. of PD / CD patients · No. of normals

Supplementary Figure S1 | Overview of transcriptomic datasets for Parkinson's and Crohn's disease. Datasets for Parkinson's disease (PD) include substantia nigra and peripheral blood. Crohn's disease (CD) datasets comprise ileal mucosa, colonic mucosa, and peripheral blood. Detailed metadata include data accession IDs, sequencing platform identifiers, dataset and tissue sources, and patient and control sample sizes. The following icons represent different tissue sources: : substantia nigra (SN); : peripheral blood; : ileal mucosa (IM); : colonic mucosa (CM).

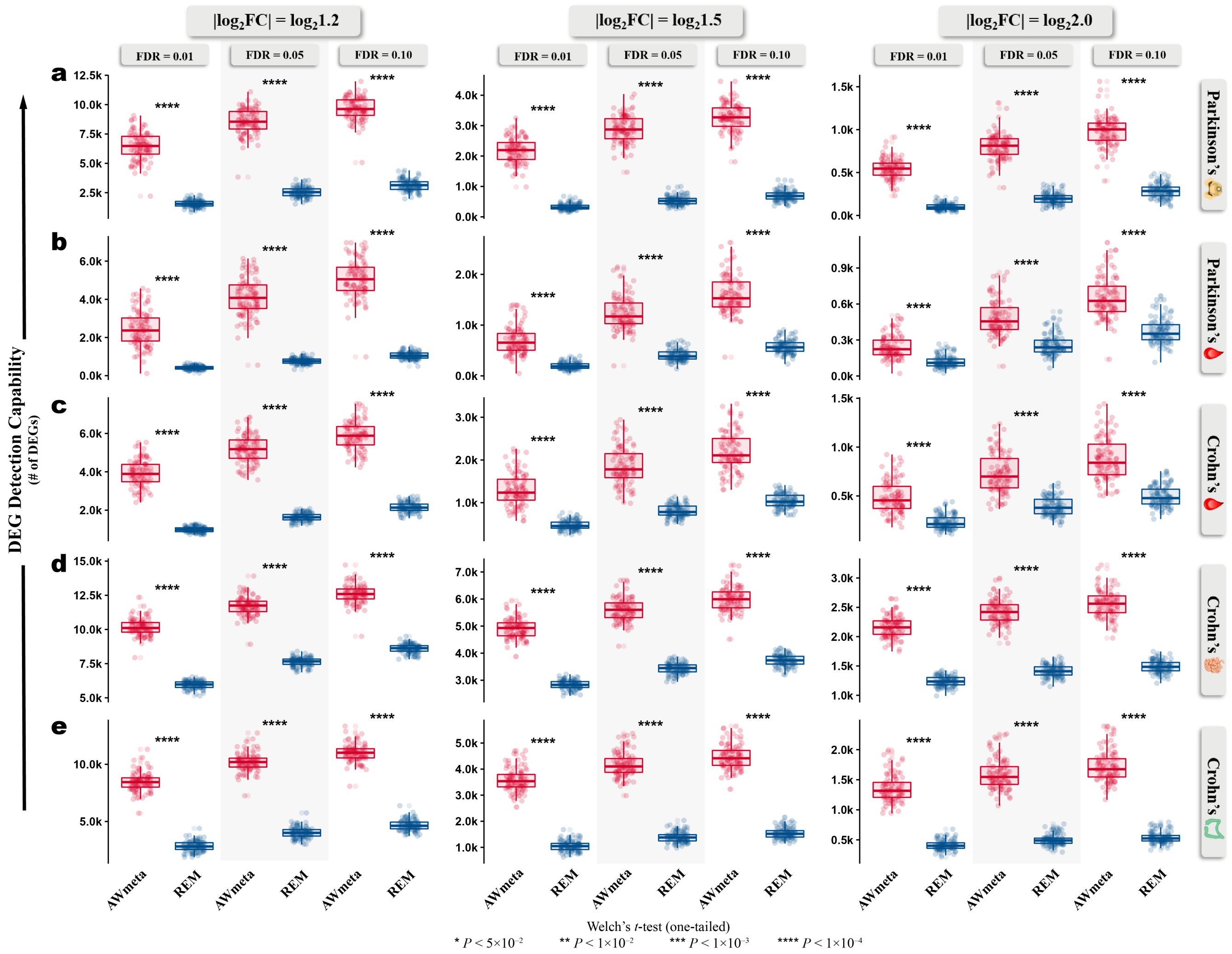

Supplementary Figure S2 | DEG detection capability evaluation across five disease tissues and diverse thresholds. (**a–e**) DEG detection capability (the number of identified DEGs) was assessed against AWmeta and REM using nine distinct thresholds, combining three corrected *P*-values (FDR) (0.01, 0.05, and 0.10) and three $\log_2$-based fold change ($|\log_2 FC|$) cutoffs ($\log_2 1.2$, $\log_2 1.5$, and $\log_2 2.0$), spanning Parkinson's substantia nigra, Parkinson's and Crohn's peripheral blood, and Crohn's ileal and colonic mucosa. Detailed description of DEG detection capability can be referred to in "2.4.1. DEG detection capability evaluation" section and Figure 1b. Statistical significance was determined using one-tailed Welch's *t*-test. Boxplot bounds show interquartile ranges (IQR), centers indicate median values, and whiskers extend to 1.5×IQR. The following icons represent different tissue sources: : substantia nigra; : peripheral blood; : ileal mucosa; : colonic mucosa.

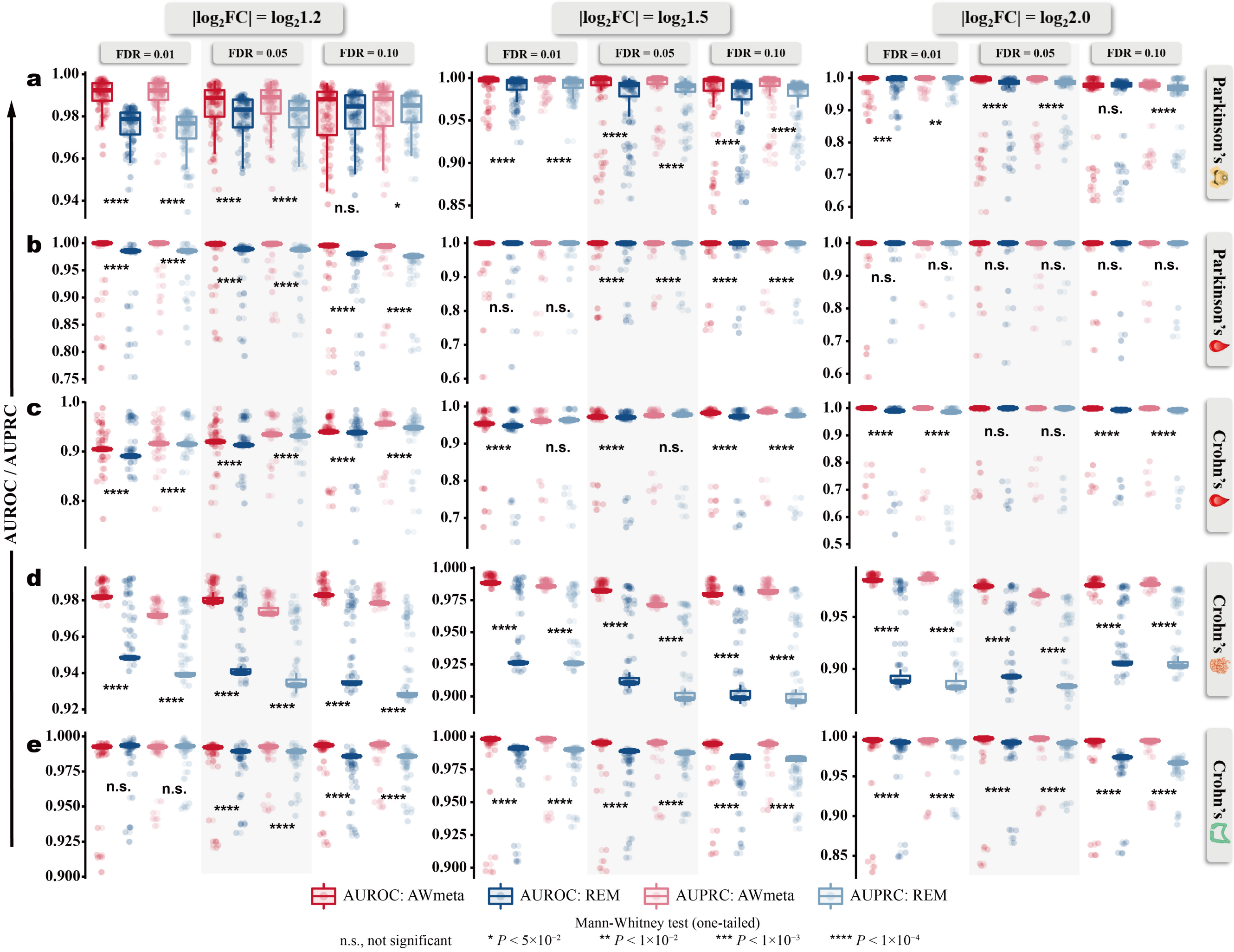

Supplementary Figure S3 | DEG discrimination evaluation across five disease tissues using minimum-permuted semi-synthetic simulation strategy. (**a**–**e**) DEG discrimination (AUROC and AUPRC) was assessed against AWmeta and REM using nine distinct thresholds, combining three corrected *P*-values (FDR) (0.01, 0.05, and 0.10) and three $\log_2$-based fold change ($|\log_2 FC|$) cutoffs ($\log_2 1.2$, $\log_2 1.5$, and $\log_2 2.0$), spanning Parkinson's substantia nigra, Parkinson's and Crohn's peripheral blood, and Crohn's ileal and colonic mucosa. Detailed description of minimum-permuted semi-synthetic simulation strategy can be referred to in "2.4.2. DEG discrimination evaluation using semi-synthetic simulation strategy" section, "3.1. AWmeta secures consistent higher-fidelity DEG identification across transcriptomic contexts of Parkinson's and Crohn's disease" section and Figure 1d–f. Statistical significance was determined using one-tailed Mann-Whitney test. Boxplot bounds show interquartile ranges (IQR), centers indicate median values, and whiskers extend to 1.5×IQR. The following icons represent different tissue sources: 🧠: substantia nigra; 🩸: peripheral blood; ileal icon: ileal mucosa; colon icon: colonic mucosa.

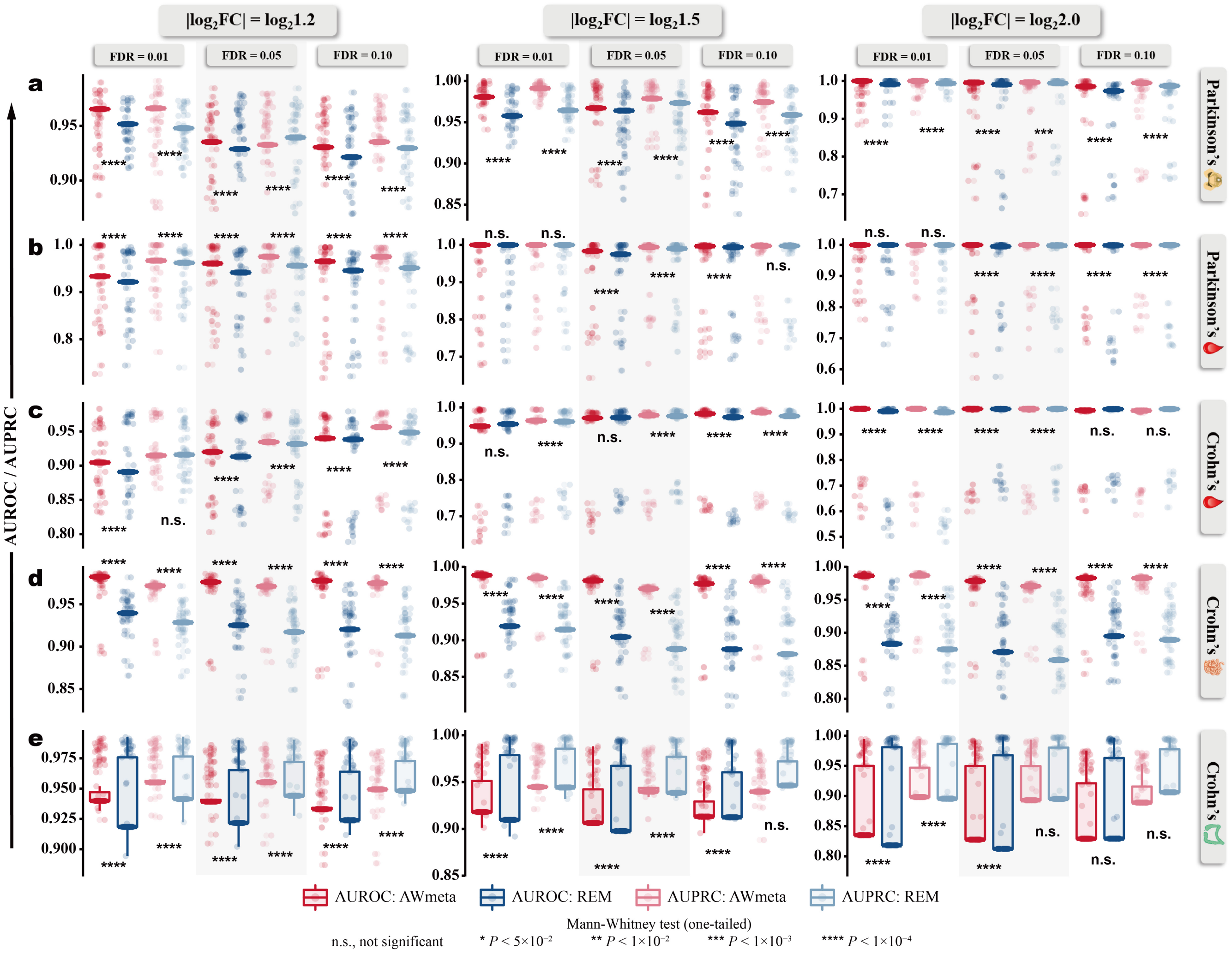

Supplementary Figure S4 | DEG discrimination evaluation across five disease tissues using median-permuted semi-synthetic simulation strategy. (**a**–**e**) DEG discrimination (AUROC and AUPRC) was assessed against AWmeta and REM using nine distinct thresholds, combining three corrected *P*-values (FDR) (0.01, 0.05, and 0.10) and three $\log_2$-based fold change ($|\log_2 FC|$) cutoffs ($\log_2 1.2$, $\log_2 1.5$, and $\log_2 2.0$), spanning Parkinson's substantia nigra, Parkinson's and Crohn's peripheral blood, and Crohn's ileal and colonic mucosa. Detailed description of median-permuted semi-synthetic simulation strategy can be referred to in "2.4.2. DEG discrimination evaluation using semi-synthetic simulation strategy" section, "3.1. AWmeta secures consistent higher-fidelity DEG identification across transcriptomic contexts of Parkinson's and Crohn's disease" section and Figure 1d–f. Statistical significance was determined using one-tailed Mann-Whitney test. Boxplot bounds show interquartile ranges (IQR), centers indicate median values, and whiskers extend to 1.5×IQR. The following icons represent different tissue sources: : substantia nigra; : peripheral blood; : ileal mucosa; : colonic mucosa.

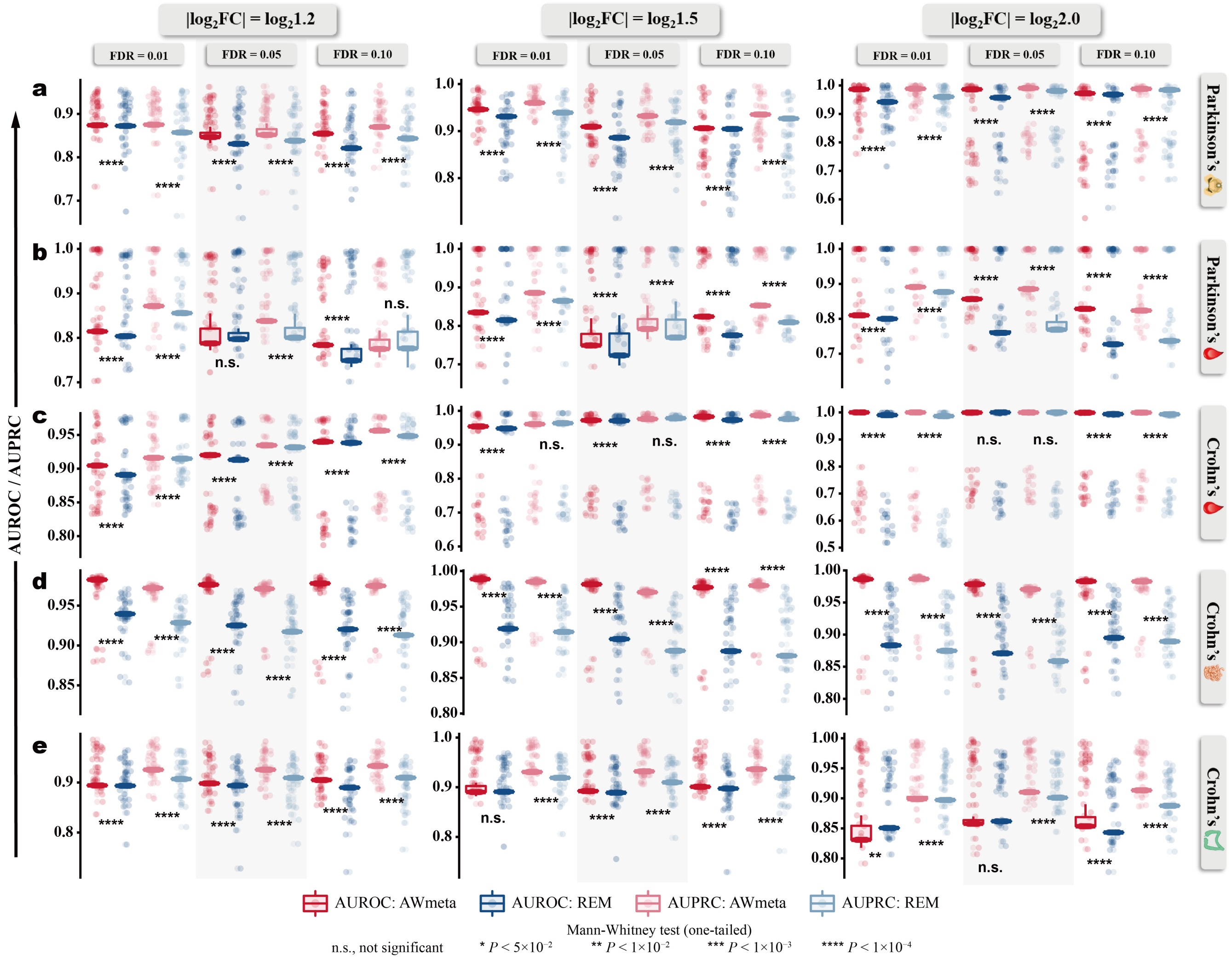

Supplementary Figure S5 | DEG discrimination evaluation across five disease tissues using maximum-permuted semi-synthetic simulation strategy. (**a**–**e**) DEG discrimination (AUROC and AUPRC) was assessed against AWmeta and REM using nine distinct thresholds, combining three corrected *P*-values (FDR) (0.01, 0.05, and 0.10) and three $\log_2$-based fold change ($|\log_2 FC|$) cutoffs ($\log_2 1.2$, $\log_2 1.5$, and $\log_2 2.0$), spanning Parkinson's substantia nigra, Parkinson's and Crohn's peripheral blood, and Crohn's ileal and colonic mucosa. Detailed description of maximum-permuted semi-synthetic simulation strategy can be referred to in "2.4.2. DEG discrimination evaluation using semi-synthetic simulation strategy" section, "3.1. AWmeta secures consistent higher-fidelity DEG identification across transcriptomic contexts of Parkinson's and Crohn's disease" section and Figure 1d–f. Statistical significance was determined using one-tailed Mann-Whitney test. Boxplot bounds show interquartile ranges (IQR), centers indicate median values, and whiskers extend to 1.5×IQR. The following icons represent different tissue sources: : substantia nigra; : peripheral blood; : ileal mucosa; : colonic mucosa.

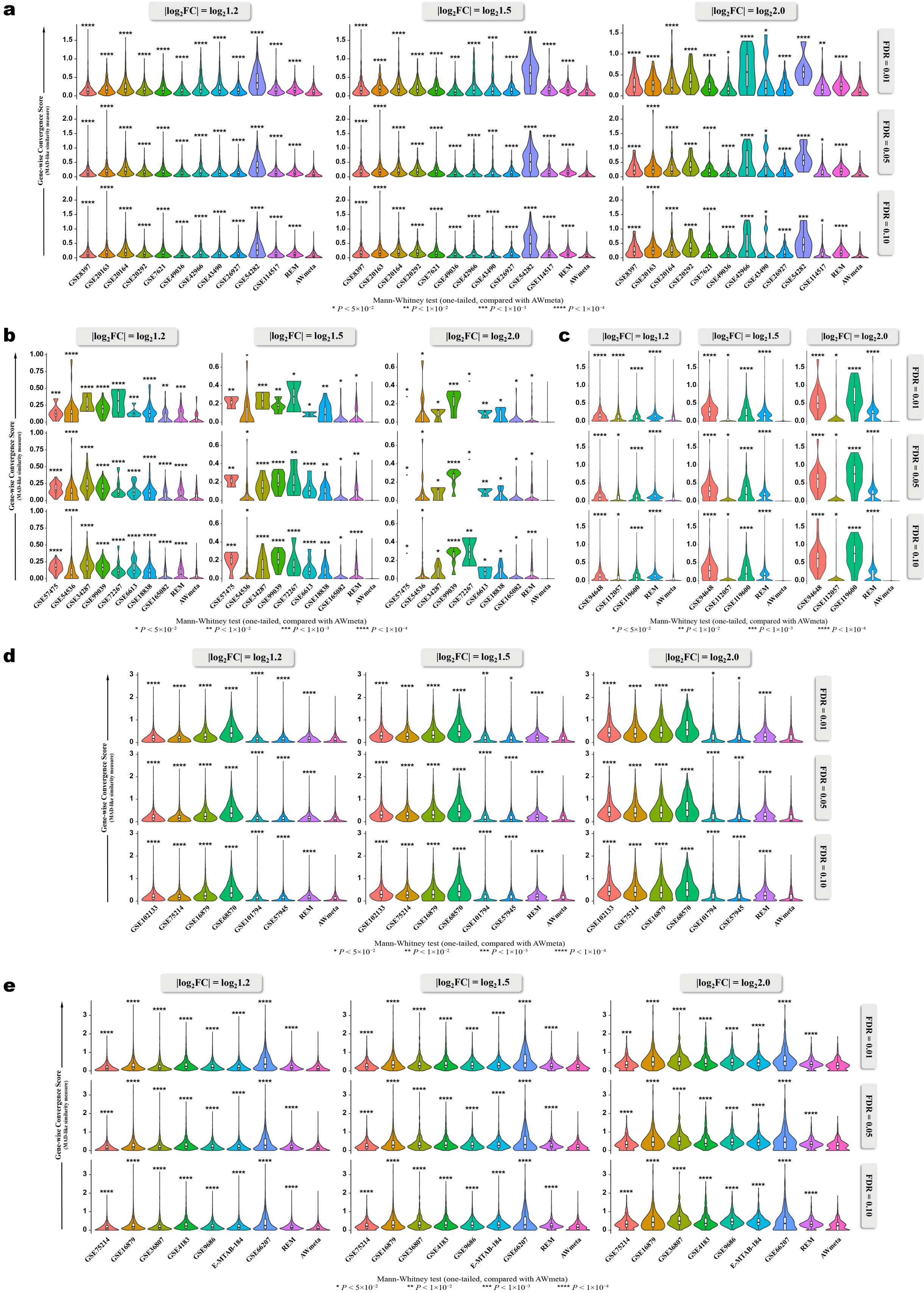
a
|log2FC| = log21.2
|log2FC| = log21.5
|log2FC| = log22.0
FDR = 0.01
FDR = 0.05
FDR = 0.10
Gene-wise Convergence Score
(MAD-like similarity measure)
GSE8397
GSE20163
GSE20164
GSE20292
GSE7621
GSE49036
GSE42966
GSE43490
GSE26927
GSE54282
GSE114517
REM
AWmeta
Mann-Whitney test (one-tailed, compared with AWmeta)
* P < 5×10−2
** P < 1×10−2
*** P < 1×10−3
**** P < 1×10−4
b
GSE57475
GSE54536
GSE34287
GSE99039
GSE72267
GSE6613
GSE18838
GSE165082
c
GSE94648
GSE112057
GSE119600
d
GSE102133
GSE75214
GSE16879
GSE68570
GSE101794
GSE57945
e
GSE36807
GSE4183
GSE9686
E-MTAB-184
GSE66207

Supplementary Figure S6 | AWmeta establishes superior DEG-wise convergence in gene differential expression meta-analysis. Considering that DEGs instead of non-DEGs are primarily involved in disease etiology, to explore whether gene-(unfiltered) and DEG-wise convergence assessment results are different, mean absolute deviation (MAD) -like similarity measure was utilized to quantify the per-DEG fold change ($|log_2FC|$) similarity among AWmeta, REM and original studies, with smaller values indicating better convergence, which demonstrates AWmeta's consistent superior DEG- with gene-wise convergence over REM and orignal studies using nine distinct thresholds, combining three corrected *P*-values (FDR) (0.01, 0.05, and 0.10) and three log_2-based fold change ($|log_2FC|$) cutoffs ($log_2 1.2$, $log_2 1.5$, and $log_2 2.0$), across five disease tissues: (**a**) Parkinson's substantia nigra, (**b**) Parkinson's peripheral blood, (**c**) Crohn's peripheral blood, (**d**) Crohn's ileal mucosa and (**e**) Crohn's colonic mucosa. For comparison purpose, results from original studies serve as reference baselines. Statistical significance of REM and baselines against AWmeta for DEG-wise convergence comparisons was tested with one-tailed Mann-Whitney test. Detailed description for MAD-like DEG-wise convergence similarity measure can be referred to in "2.4.3. Gene-wise convergence assessment for gene differential expression meta-analysis" section, "3.2. AWmeta establishes superior gene- and study-wise convergence in gene differential expression of Parkinson's and Crohn's disease" section and Figure 2a. Boxplot bounds show interquartile ranges (IQR), centers indicate median values, and whiskers extend to 1.5×IQR. The following icons represent different tissue sources: : substantia nigra; : peripheral blood; : ileal mucosa; : colonic mucosa.

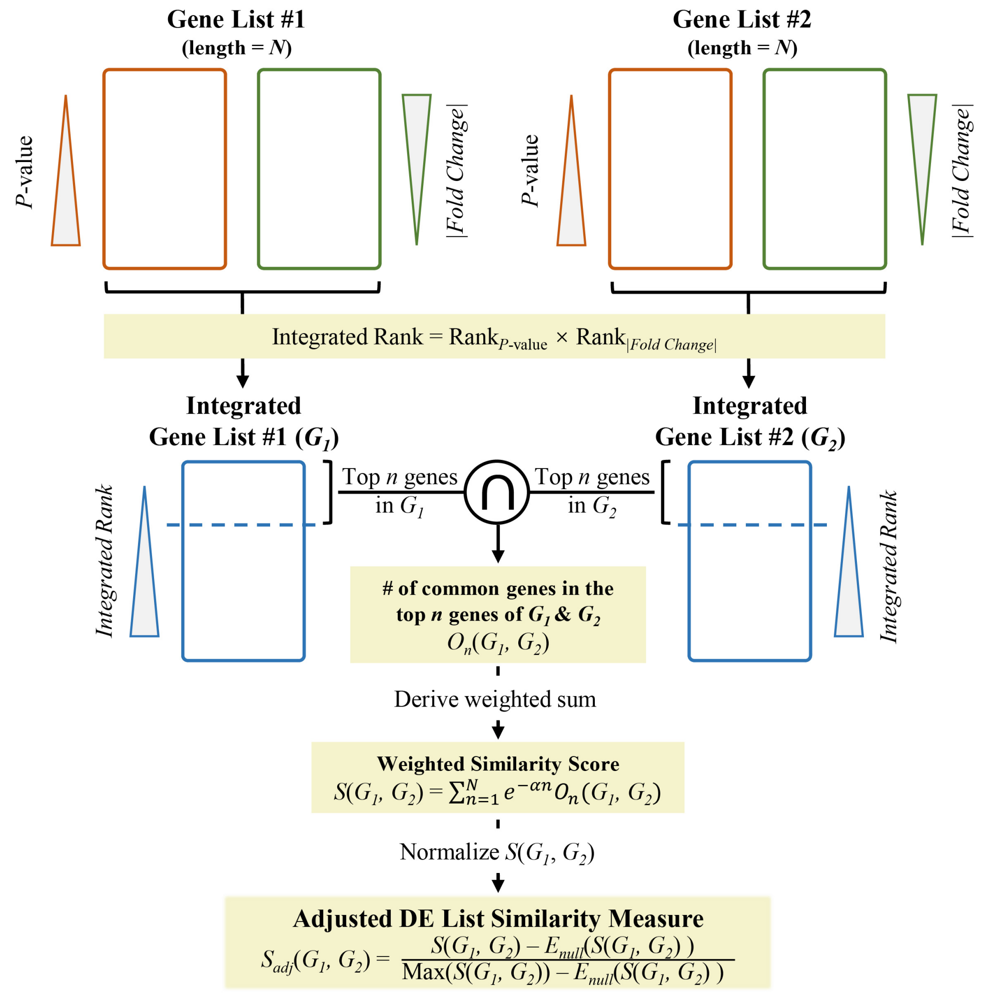

Supplementary Figure S7 | Conceptual schematic of adjusted DE list similarity measure. The methodological details reside in "2.4.4. Study-wise convergence assessment for gene differential expression meta-analysis" section.

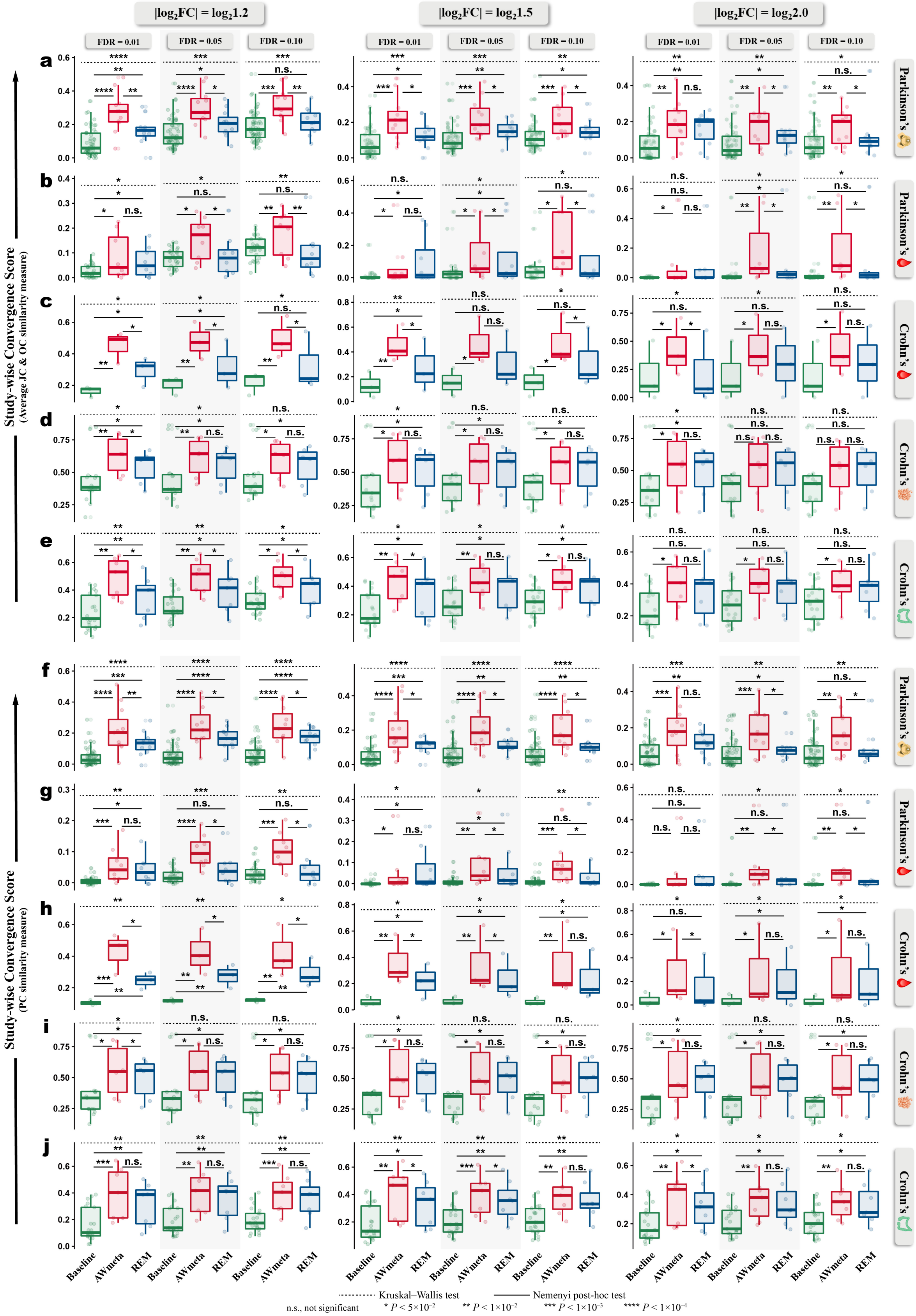

|log2FC| = log21.2
|log2FC| = log21.5
|log2FC| = log22.0
FDR = 0.01
FDR = 0.05
FDR = 0.10
Study-wise Convergence Score
(Average JC & OC similarity measure)
Study-wise Convergence Score
(PC similarity measure)
Parkinson's
Crohn's
Baseline
AWmeta
REM
Kruskal–Wallis test
Nemenyi post-hoc test
n.s., not significant
* P < 5×10−2
** P < 1×10−2
*** P < 1×10−3
**** P < 1×10−4

Supplementary Figure S8 | AWmeta maintains robust superior study-wise convergence in gene differential expression meta-analysis across diverse thresholds. (**a–j**) To dissect whether set-theory-based similarity-derived study-wise convergence assessment results vary with diverse DEG thresholds, we used nine distinct thresholds, combining three corrected *P*-values (FDR) (0.01, 0.05, and 0.10) and three $\log_2$-based fold change ($|\log_2 FC|$) cutoffs ($\log_2 1.2$, $\log_2 1.5$, and $\log_2 2.0$), to benchmark study-wise convergence, which showcases AWmeta maintains robust superior study-wise convergence in gene differential expression across diverse thresholds over REM and baselines in five disease tissues, both by means of (**a–e**) the average of Jaccard (JC) and overlap coefficient (OC) and (**f–j**) phi coefficient (PC). For comparison purpose, results from original studies serve as reference baselines. Overall study-wise convergence differences among AWmeta, REM and baselines were tested by Kruskal–Wallis test, followed by Nemenyi post-hoc test for pairwise comparisons. Details for these two study-wise convergence similarity measures appear in "2.4.4. Study-wise convergence assessment for gene differential expression meta-analysis" section and Figure 2h–j. Boxplot bounds show interquartile ranges (IQR), centers indicate median values, and whiskers extend to $1.5\times$IQR. The following icons represent different tissue sources: : substantia nigra; : Peripheral blood; : ileal mucosa; : colonic mucosa.
